# Anti-Inflammatory Effects and Metabolomic Analysis of Ilex Rotunda Extracted by Supercritical Fluid Extraction

**DOI:** 10.3390/ijms252211965

**Published:** 2024-11-07

**Authors:** Duc Dat Le, Young Su Jang, Vinhquang Truong, Thientam Dinh, Thinhulinh Dang, Soojung Yu, Mina Lee

**Affiliations:** 1 College of Pharmacy and Research Institute of Life and Pharmaceutical Sciences, Sunchon National University, 255 Jungangno, Suncheon 57922, Jeonnam, Republic of Korea; ddle@scnu.ac.kr (D.D.L.); quangvtruong00@gmail.com (V.T.); thientamm.2001@gmail.com (T.D.); dangnhulinh02051998@gmail.com (T.D.); 2 Nano Bio Research Center, Jeonnam Bio Foundation, Jangseong 57248, Jeonnam, Republic of Korea; ysjang@jbf.kr; 3 Department of Natural Cosmetics Science, Graduate School, Sunchon National University, 255 Jun-Gangno, Suncheon 57922, Jeonnam, Republic of Korea; ysj1997s@naver.com

**Keywords:** *Ilex rotunda*, inflammatory cytokines, IL-2 production, mass dereplication, RAW264.7, Jurkat T cells

## Abstract

*Ilex rotunda* is a famous medicinal plant with many ethnopharmacological uses. It is traditionally employed for treating inflammation and cardiovascular diseases. In this study, we established green technology to extract the leaves and twigs of *I*. *rotunda*. The obtained extracts and their fractions were evaluated for their anti-inflammatory potential. In cytokine assays, the extract, *n*-hexane (H)*,* methylene chloride (MC), and EtOAc (E) fractions of the twigs of *I*. *rotunda* significantly inhibited lipopolysaccharide (LPS)-induced nitric oxide (NO), interleukin (IL)-6, and tumor necrosis factor (TNF)-α production in RAW264.7 macrophages. Furthermore, the extract, H, and MC fractions of the leaves of *I*. *rotunda* modulated cytokine expression by downregulating LPS-induced NO, IL-6, and TNF-α production in RAW264.7 macrophages. Western blotting analysis revealed that the extracts and fractions of the leaves and twigs of *I*. *rotunda* inhibited inflammatory cytokines by inactivating nuclear factor kappa B (NFκB) action by reducing the phosphorylation of transcript factor (p65) and nuclear factor-kappa B inhibitor alpha (IκBα) degradation, or by inactivating mitogen-activated protein kinase (MAPK) through the p38 or ERK signaling pathways via the active ingredients of the leaves and twigs of *I*. *rotunda*. Ultra-high-resolution liquid chromatography–Orbitrap mass analysis (UHPLC–ESI-Orbitrap-MS/MS)-based molecular networking, in cooperation with social open platform-guided isolation and dereplication, led to the identification of metabolites in this plant. Our findings indicate that the leaves and twigs of *I*. *rotunda* could be promising candidates for developing therapeutic strategies to treat anti-inflammatory diseases.

## 1. Introduction

*Ilex rotunda* Thum. belongs to the *Ilex* genus of Aquifoliaceae. It is distributed throughout East Asia. As a well-known medicinal plant, it has many ethnopharmacological uses. It is traditionally used for treating rheumatism, traumatic injury, inflammation, and cardiovascular diseases [1,2]. Its chemical compositions have a wide range of pharmacological effects, including antitumor, antibacterial, antioxidative, anti-hyperlipidemic, anti-hyperglycemia, and metabolic activities [3,4]. Previous studies have demonstrated that its extracts may treat inflammatory diseases and impaired liver function by downregulating the expression of cytokines and transcription factors and decreasing inflammatory reactions [5]. In contrast, some active compounds such as syringin, pedunculoside, and rotundic acid have shown significant effects in in vitro and in vivo studies [6,7,8].

Inflammation is a defense response in our body to harmful signals and tissue damage [9,10]. There are many mediators and cytokines that are generated during the inflammatory process, and they have significant impacts on human health. They are released by inflammation-inducing pathogen-associated molecular patterns, including highly conserved structures such as LPS-activated immune cells [11], and may induce further cytokine production when activated through different signaling cascades [12,13]. As a result, sudden cytokine storms may be released to upregulate the inflammatory process [14]. However, an uncontrolled inflammatory response may lead to a vast continuum of disorders and cause side-effects in the body [15]. Herbal medicines and their secondary metabolites are often employed and developed as medications to treat inflammatory diseases with minimum adverse effects [16]. Therefore, natural products can have high pharmacological activity and low-toxicity properties and could serve as potential resources for the development of promising anti-inflammatory drug candidates [17].

Green extraction has attracted great attention because of its high potential for application in extracting target components or because it can reduce the use of solvents or chemicals known to be hazardous to human health [18]. The supercritical fluid extraction (SFE) method is known to be environmentally friendly and versatile. It can be employed to prepare large-scale batches for industrial applications [19]. This extraction method provides many advantages, such as substantially saving costs and time during experiments. Furthermore, SFE may be optimized by adjusting the parameters associated with solvent selection, according to the desired extraction of non-polar or polar compounds. Thus, SFE is considered a green chemistry technique that can solve some of the important drawbacks associated with conventional methods that use toxic solvents extensively and require high amounts of energy in the pharmaceutical industry [20].

In this study, SFE technology was used to extract *I*. *rotunda* leaves and twigs for the first time. The obtained extracts and their fractions showed anti-inflammatory effects against cytokines and mediators in in vitro assays. Active fractions were also assessed for their activities through Western blotting. Active fractions also mediate the MAPK and NFκB signaling pathways. To investigate the chemical compositions of twig and leaf extracts of *I*. *rotunda* and their fractions, liquid chromatography–tandem mass spectrometry (LC–MS/MS) dereplication was performed to find the metabolites within the total extracts and their fractions, using the Global Natural Products Social (GNPS) webtool, MS-Dials, and public databases.

## 2. Results

### 2.1. Supercritical Fluid Extraction (SFE)

In this study, SFE with elevated pressure and EtOH mixed with CO_2_ as co-solvent was used to extract thermolabile components from the leaves and twigs of *I. rotunda* at 50 °C and a pressure of 400 bar. Under the above extract conditions, EtOH mixed with CO_2_ was transformed into a supercritical state that could enter easily into the cells of the dried leaves and twigs to remove secondary metabolites out of the leaf and twig materials. The leaf and twig extracts were collected after removing the EtOH. Almost all the extracts were successfully partitioned into different fractions according to the polarity between co-partner solvents such as n-hexane, methylene chloride, EtOAc, and *n*-BuOH after suspending them in distilled water. Finally, five distinct fractions, H, MC, E, B, and W, were collected and used for further assays.

### 2.2. Antioxidative Effects of Extracts and Fractions

**Figure 1:**
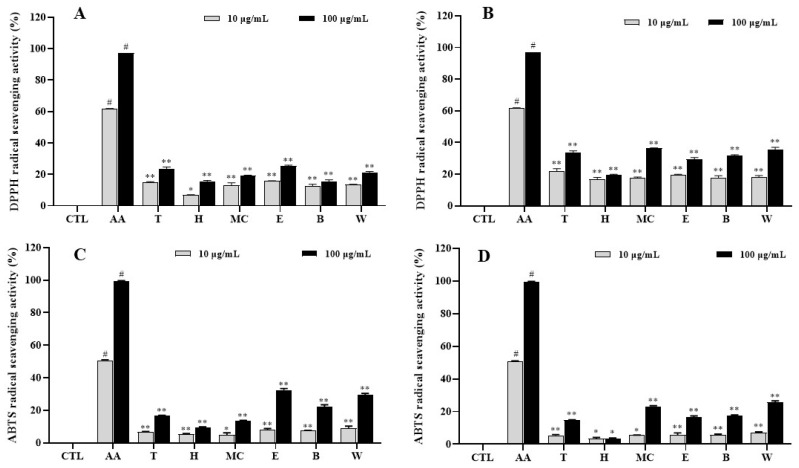
Antioxidative effects of leaf extract and fractions (**A**,**C**) and twig extract and fractions (**B**,**D**) were accessed by scavenging activity of DPPH and ABTS radicals. Samples were assayed at 10 and 100 µg/mL in triplicates. Values are expressed as means ± SD. ^#^ *p* < 0.05 vs. non-treated group (CTL). Differences were significant at * *p* < 0.05 and ** *p* < 0.01 compared to positive control (Ascorbic acid, AA).

To assess the antioxidative potential of *I*. *rotunda*, its leaf and twig extracts and their fractions were tested for their ability to scavenge DPPH and ABTS radicals. The DPPH assays of all samples from leaves revealed that the E fraction exhibited the strongest radical scavenging activity with an inhibition rate of approximately 25.4%, while the hexane fraction showed the weakest activity among leaf samples at 100 µg/mL (Figure 1A). In contrast, almost all twig samples exhibited higher radical scavenging activities compared to leaf samples. In particular, the MC, E, B, and W fractions displayed good radical scavenging activities ranging from 36.5% to 29.4%, and the hexane fraction presented the lowest activity of all samples at the same tested concentration. Similar trends were observed for leaf and twig samples at a concentration of 10 µg/mL (Figure 1B).

However, leaf extract and fractions demonstrated stronger activities than those derived from twigs in the ABTS assay. Among all samples, the E, B, and W fractions from leaves exhibited higher ABTS radical scavenging activities with percentages of 32.4%, 22.1%, and 29.6%, respectively (Figure 1C). For the twig samples, the MC and W fractions displayed significant radical scavenging activities of 23.2% and 25.7%, respectively, at the tested concentration of 100 µg/mL (Figure 1D).

### 2.3. Effects of Leaf and Twig Extracts and Their Fractions on Cell Viability

**Figure 2:**
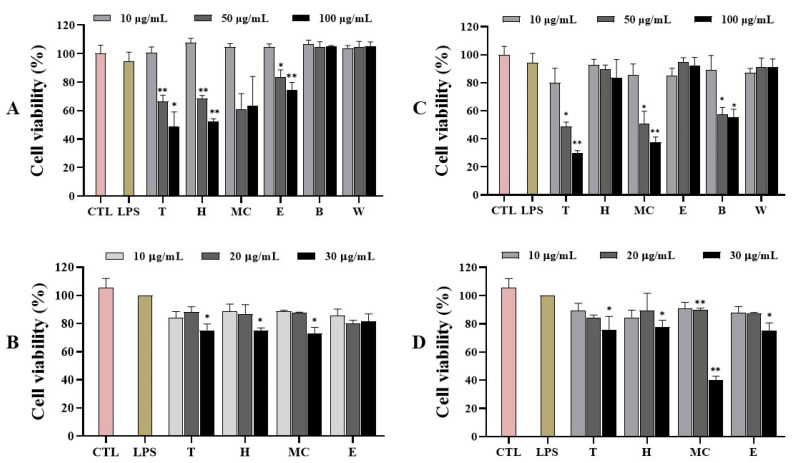
Cytotoxic effects of leaf (**A**,**B**) and twig (**C**,**D**) extracts and their fractions on cell survival at different concentrations. Cell viability was assessed using the MTT assay. RAW264.7 cells were treated with LPS or samples in a dose-dependent manner at concentrations of 10, 20, 30, 50, and 100 µg/mL and repeated three times. Values are expressed as means ± SD. Differences were significant at * *p* < 0.05 and ** *p* < 0.01, compared to the control (CTL).

To evaluate cytotoxicity in RAW264.7 macrophages, leaf and twig extracts along with their fractions were initially tested at concentrations of 10, 50, and 100 µg/mL using the 3-(4,5-dimethylthiazol-2-yl)-2,5-diphenyl-2H-tetrazolium bromide (MTT) assay. Among all samples tested, leaf extracts and fractions H, MC, and E showed some toxic effects on cell survival at 50 and 100 µg/mL (Figure 2A). Additionally, the twig extracts and fractions MC, E, and B affected cell viability at 50 and 100 µg/mL (Figure 2B). However, none of the samples affected cell viability at the tested concentration of 10 µg/mL. To assess the impacts of these active extracts and fractions on RAW264.7 cells’ viability, they were then tested at low concentrations of 10, 20, and 30 µg/mL. The results indicated that leaf extracts and fractions did not have seriously toxic effects at the tested concentrations on the cell viability of RAW264.7, with cell survival exceeding 77.5% in a dose-dependent manner (Figure 2C). Among the twig samples, the MC fraction exhibited a toxic effect at concentrations of 30 µg/mL, with cell survival being below 40.3% (Figure 2D). All non-toxic samples had no impact on cell morphological change.

### 2.4. Anti-Inflammatory Effects

**Figure 3:**
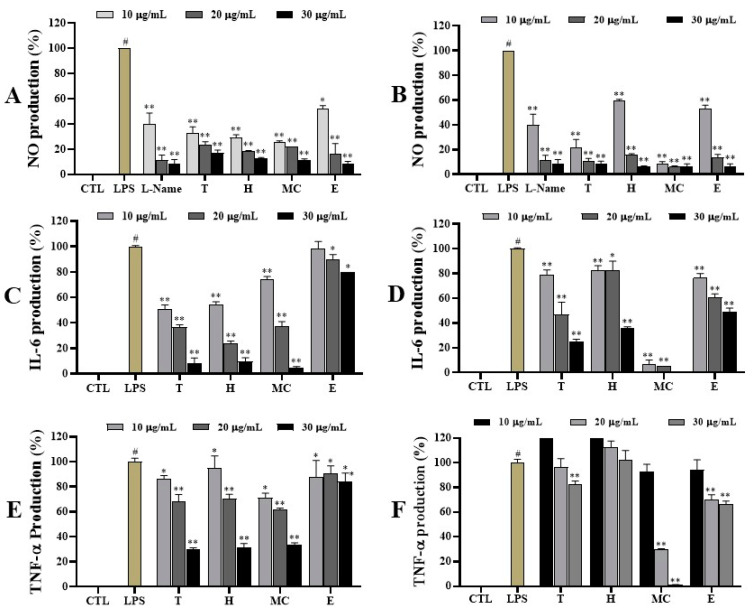
The inhibitory effects of the leaf and twig extracts and their fractions on NO (**A**,**B**), IL-6 (**C**,**D**), and TNF-α (**E**,**F**) production induced by LPS-activated RAW264.7 cells. Cells were pretreated with tested samples (10, 20, and 30 µg/mL) for 1 h and then stimulated with LPS (5 ng/mL) for 24 h. NO, IL-6, and TNF-α production in the culture media were quantified using the Griess assay and an enzyme immunoassay (EIAISA) kit, respectively. ^#^ *p* < 0.05 vs. non-treated group (CTL). Differences were significant at * *p* < 0.05 and ** *p* < 0.01 compared to controls.

To assess the anti-inflammatory effects of leaf and twig extracts and their fractions, we evaluated the inhibitory effects of the above extracts and fractions on inflammatory mediators and cytokines. In an NO assay with samples tested at 30 µg/mL, leaf extracts and fractions significantly inhibited NO production in LPS-stimulated RAW264.7 cells. Notably, E fraction exhibited the strongest inhibitory effect (inhibition rate of 91.2%), being the same as those of the positive control (91.2%) in LPS-induced NO production. The H fraction, MC fraction, and leaf extract also significantly reduced NO levels in LPS-activated RAW264.7 cells, with inhibition rates of 88.5%, 87.7%, and 82.0%, respectively. At a concentration of 20 µg/mL, E, H, and MC fractions and the extracts substantially decreased NO production in LPS-stimulated RAW264.7 cells. At a concentration of 10 µg/mL, the MC fraction displayed the most inhibitory effect, followed by the H fraction, leaf extract, and E fraction (Figure 3A). Concerning the impact of twig extracts and fractions at 30 µg/mL, all samples significantly blocked NO production of LPS-activated macrophage in a dose-dependent manner. At a concentration of 30 µg/mL, E, MC, and H fractions and twig extracts showed strong inhibitory effects, with inhibition rates ranging from 94.0% to 91.8%, surpassing the positive control (L-NAME, 91.2%). At 20 µg/mL, the twig extracts and fractions also displayed significant inhibitory effects on NO production. At 10 µg/mL, twig extracts and MC fraction demonstrated notable effects, with inhibition rates of 91.2% and 78.1%, respectively, while the H and E fractions showed weaker effects under the same experimental conditions (Figure 3B).

At 30 µg/mL, the leaf extract and fractions H and MC strongly inhibited IL-6 production in LPS-activated RAW264.7 cells, with inhibition rates of 92.1%, 90.5%, and 95.6%, respectively. At 20 µg/mL, the H fraction significantly inhibited IL-6 production (76.1%). The MC fraction and leaf extract also downregulated IL-6 production, showing inhibition rates of 62.8% and 63.5%, respectively. At 10 µg/mL, the H and MC fractions exhibited moderate inhibitory effects, whereas the leaf extract had a weak inhibitory effect. The E fraction showed weak inhibition against IL-6 production at all concentrations (Figure 3C). Similarly, the twig extract and fractions also inhibited IL-6 induced by LPS in RAW264.7 cells in different ways. The MC fraction markedly inhibited IL-6 production at all concentrations, with inhibition rates ranging from 93% to 99.8%. At 30 µg/mL, the twig extract and H fraction significantly moderated IL-6 production with inhibition rates of 75.2% and 64.5%, respectively, compared to a sample-free control in LPS-stimulated RAW264.7 cells. At 10 and 20 µg/mL, twig extract and H and E fractions demonstrated moderate inhibitory effects on IL-6 production compared to LPS-treated RAW264.7 cells without sample addition (Figure 3D).

In the TNF-α assay, the leaf extract and H fraction suppressed TNF-α production induced by LPS in RAW264.7 cells at a concentration of 30 µg/mL, significantly inhibiting TNF-α production, with inhibition rates ranging from 69.9% to 68.5% compared to a sample-free control in LPS-stimulated RAW264.7 cells. At 10 and 20 µg/mL, the leaf extract and H and MC fractions showed weak inhibitory activities against TNF-α production (Figure 3E). Additionally, the E fraction had a weak inhibitory effect on TNF-α production at all tested concentrations. Among the twig extract and fractions, the MC fraction strongly suppressed TNF-α production at 20 and 30 µg/mL in LPS-activated RAW264.7 cells, with inhibition rates of 70.5% and 98.6%, respectively (Figure 3F). Other samples did not show significant inhibition at tested concentrations.

### 2.5. MNM Extract and Fractions Regulate iNOS and COX-2 Expression

**Figure 4:**
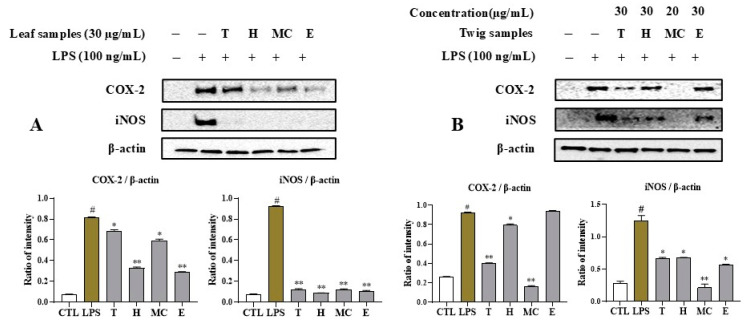
Western blot analysis of the effects of leaf extract and fractions (**A**) and twig extract and fractions (**B**) on iNOS, COX-2 expression levels. LPS-induced RAW264.7 macrophages were pretreated with samples at concentrations of 30 μg/mL and incubated for 6 h. Total proteins were then isolated, separated by SDS-PAGE, and immunoblotted using specific p-p65, p-IκBα, and IκBα antibodies. β-Actin served as an internal control. Relative optical density ratio vs. β-actin or total form was determined using a densitometric analysis program (Bio-Rad Quantity One Software, version 4.6.3 (Basic), Bio-Rad Laboratories Inc., Hercules, CA, USA), normalized to internal control. ^#^ *p* < 0.05 vs. non-treated group (CTL). Differences were significant at * *p* < 0.05, ** *p* < 0.01 compared to non-sample in LPS stimulation.

To investigate the inhibition mode of the active fractions on inflammation, these active samples were further evaluated for their ability to suppress iNOS and COX-2 expression. The leaf extract and fractions markedly reduced iNOS expression levels compared to the non-treated sample with LPS stimulation without affecting β-actin at 30 µg/mL. Furthermore, the H and E fractions of the leaf extract inhibited the expression level of the COX-2 enzymatic protein without affecting β-actin expression at a concentration of 30 µg/mL (Figure 4A). Among the twig samples, the MC fraction strongly downregulated both iNOS and COX-2 expression levels without affecting β-actin expression at 20 µg/mL. The H and E fractions and twig extract significantly reduced iNOS protein expression level at 30 µg/mL. The twig extract also significantly decreased COX-2 expression. Other fractions displayed weak activities at 30 µg/mL (Figure 4B). None of the samples affected β-actin expression under experimental conditions.

### 2.6. Leaf and Twig Extracts and Their Fractions Downregulate LPS-Induced NFκB Activation in RAW264.7 Macrophages

**Figure 5:**
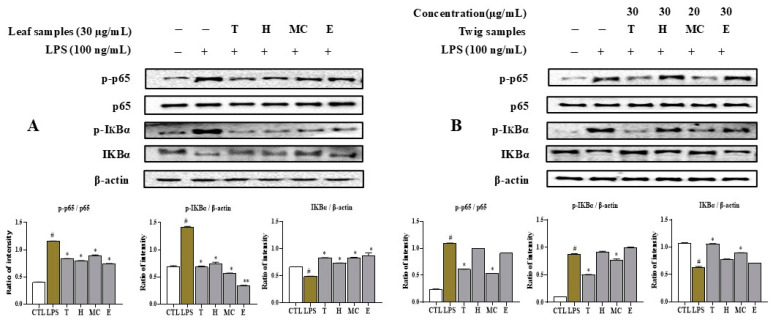
Inhibitory effects of leaf extract and fractions (**A**) and twig extract and fractions (**B**) on LPS-induced NF-κB signaling pathway in RAW264.7 macrophages. Cells were pretreated with MC fraction from twigs (20 μg/mL) and other samples (30 μg/mL) for 1 h, followed by LPS stimulation for 15 min. Proteins were isolated, separated using SDS-PAGE, and immunoblotted with antibodies specific to p-p65, p-IκBα, IκBα. β-actin was served as internal control. Relative optical density ratio versus β-actin or the total form was determined using densitometric analysis software (Bio-Rad Quantity One Software, version 4.6.3 (Basic), Bio-Rad Laboratories Inc., Hercules, CA, USA) and normalized against internal control. ^#^ *p* < 0.05 vs. non-treated group (CTL). Differences were significant at * *p* < 0.05, ** *p* < 0.01, compared to non-sample under LPS stimulation.

Western blotting assays were conducted to explore whether the NF-κB pathway mediated the effects of extracts and fractions from leaves and twigs of *I*. *rotunda* in terms of inhibiting the inflammatory response by measuring NF-κB and IκBα protein levels. As shown in Figure 5, the phosphorylation levels of p65 and IκBα were significantly increased by LPS stimulation. However, their expression levels were suppressed upon pretreatment with extract and H, MC, and E fractions of *I*. *rotunda* leaves in a dose-dependent manner. A similar effect was observed with the use of extract and fractions (H, MC, and E) from twigs of *I*. *rotunda* as a pretreatment in the presence of LPS in RAW264.7 cells. Among them, the MC fraction and twig extract significantly reduced the phosphorylation levels of p65 compared to the control with only LPS stimulation. The H and E fractions had weaker inhibition effects on p-p65 expression levels compared to LPS stimulation without sample adjustment. The MC and twig extract effectively reduced the expression levels of p-IκBα compared to the control without any treatment after LPS stimulation, while H and E fractions did not significantly affect p-IκBα expression (Figure 5). None of the tested samples significantly influenced β-Actin expression under the experimental conditions.

### 2.7. MNM Extracts and Fractions Mediate LPS-Induced MAPK Activation in RAW264.7 Cells

**Figure 6:**
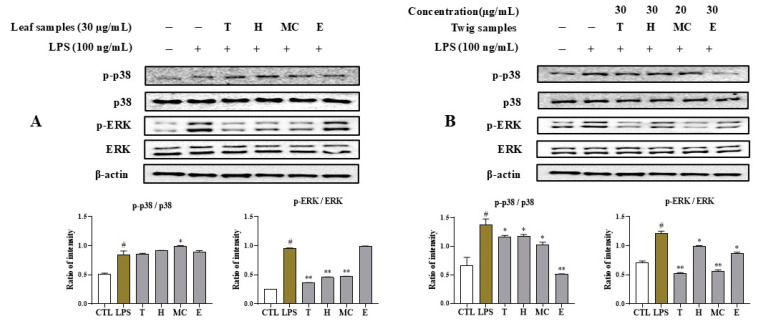
The inhibitory effects of leaf extract and fractions (**A**) and twig extract and fractions (**B**) on the LPS-induced MAPK signaling pathway in RAW264.7 macrophages. After pretreating cells with the MC fraction of twigs (20 μg/mL) and other samples (30 μg/mL) for 1 h, cells were stimulated with LPS for 15 min. Total proteins were then isolated, separated by SDS-PAGE, and immunoblotted using specific antibodies for p-p38, p-ERK, and ERK. β-Actin was served as an internal control. The relative optical density ratio vs. β-actin or total form was determined using a densitometric analysis program (Bio-Rad Quantity One Software, version 4.6.3 (Basic), Bio-Rad Laboratories Inc., Hercules, CA, USA) normalized to the internal control. ^#^ *p* < 0.05 vs. non-treated group (CTL). Differences were significant at * *p* < 0.05 and ** *p* < 0.01 compared to the control in LPS stimulation.

To investigate the effects of the above active samples on MAPK pathway, a Western blot analysis was performed. The expression levels of p-p38 and p-ERK increased following LPS stimulation. Treatment with these samples mediated their expression levels. Pretreatment with leaf extract, H, and MC fractions suppressed the phosphorylation level of ERK. However, the E fraction and leaf sample did not affect the p-ERK or p-p38 expression level (Figure 6A). Among twig samples, the E fraction remarkedly downregulated the phosphorylation of p38 compared to the control (no sample treatment with LPS stimulation). The MC fraction had a weak inhibitory effect on p-p38 protein expression. Additionally, the twig extract and fraction reduced the phosphorylation of ERK protein relative to the control (LPS stimulation without the addition of samples), displaying more pronounced effects than H and E fractions (Figure 6B). None of the samples affected the β-actin expression level under the tested conditions.

### 2.8. MNM Mediates CD3/CD28-Induced IL-2 Production in Jurkat T Cells

**Figure 7:**
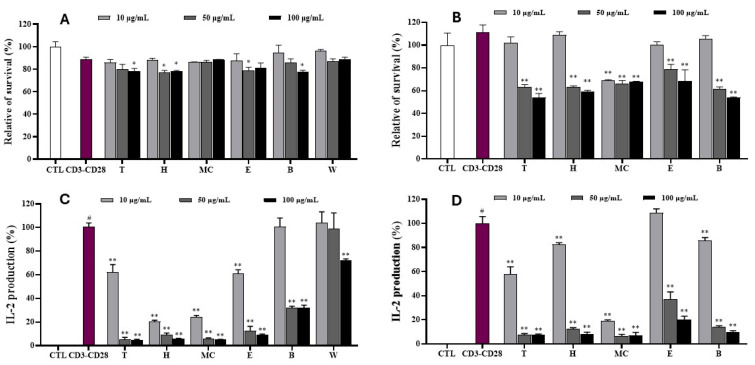
The cell viability (**A**,**B**) and inhibitory effects of leaf and twig extracts and their fractions on IL-2 production (**C**,**D**) induced by CD3-CD28 Jurkat T cells. Cells were pretreated with the samples (10, 50, and 100 µg/mL) for 1 h and subsequently stimulated with CD3-CD28 (7 µg/mL and 2 µg/mL CD3 and CD28, respectively) for 24 h. IL-2 production in the culture media was quantified using an enzyme-linked immunosorbent assay (ELISA) kit. ^#^ *p* < 0.05 vs. non-treated group (CTL). Differences were significant at * *p* < 0.05 and ** *p* < 0.01 compared to the non-sample control in CD3-CD28 stimulation.

The cytotoxic effects of leaf and twig extracts and their fractions on Jurkat T cells were evaluated using the MTT assay. As shown in Figure 7, the leaf extract and its fractions did not significantly affect the viability of T cells. However, the twig extract and its fractions exhibited toxic effects at concentrations of 50 and 100 µg/mL. Consequently, the leaf extract and its fractions were employed to investigate their influence on IL-2 production at 10, 50, and 100 µg/mL. The twig extract and its fractions were tested at 10 µg/mL. The results indicated that IL-2 production was induced by CD3/CD28 co-stimulation. After pretreatment with leaf extract and fractions, IL-2 production was dramatically regulated in a concentration-dependent manner. Notably, the leaf extract and H, MC, and E fractions strongly inhibited IL-2 production in CD3/CD28-stimulated T cells at concentrations of 50 and 100 µg/mL. Similarly, the B fraction significantly suppressed IL-2 production at the same tested conditions. At 10 µg/mL, the leaf extract and H and MC fractions significantly reduced IL-2 production in CD3/CD28-stimulated T cells compared to the untreated control.

However, the E fraction exhibited a weak inhibitory effect on IL-2 production induced by CD3/CD28-stimulated T cells. Among the twig samples tested at 10 µg/mL, twig extract and B fraction exhibited weak inhibitory effects on IL-2 production (Figure 7).

### 2.9. Phytochemical Profiling and Prediction of Compounds Using Open-Source Tools

**Figure 8:**
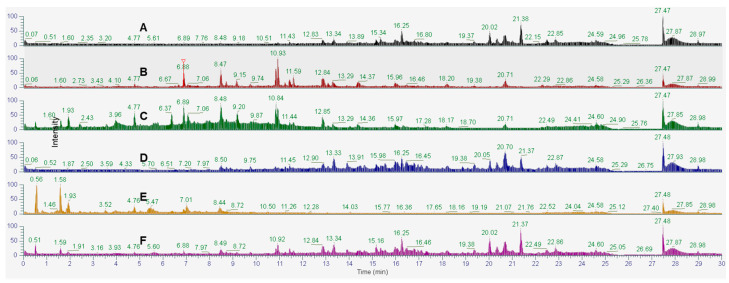
The chromatograms of H (A, black), E (B, red), B (C, green), MC (D, blue), and W fractions (E, yellow) and extracts (F, magenta) of leaves of *I*. *rotunda* detected in TIC for negative ion mode.

**Figure 9:**
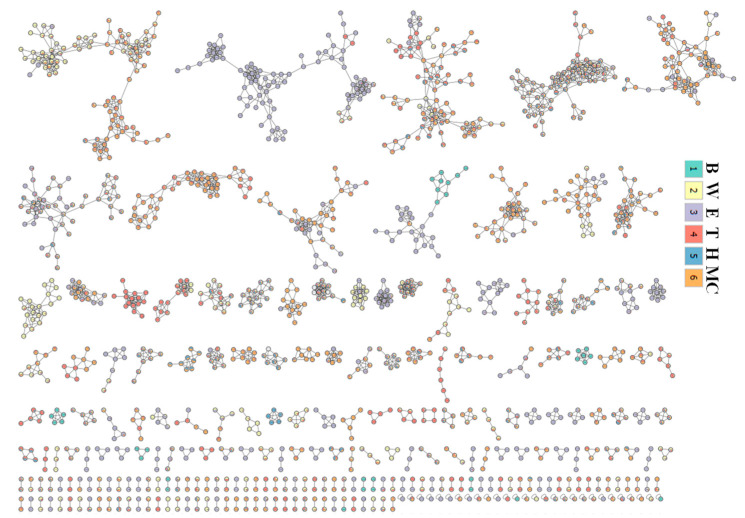
Feature-based molecular network of extract (Red) and fractions [H: Blue; MC: Orange; E: Violet; B: Dark Green; W: Yellow] of twigs (GNPS molecular network with job ID = 96ad1b5bbed84c8bb073d8c882f3897b) of *I*. *rotunda*.

Extracts and fractions of leaves and twigs of *I*. *rotunda* were analyzed by a UHPLC-Q-Orbitrap HRMS system to build representative ion chromatograms detected with TIC mode (Figure 8, Figure 9 and Figure S1, Supplementary Materials). As shown in these figures, chromatograms showed good detection of peaks with a good resolution. Among them, the MC fractions of leaf and twig extracts displayed the most abundant peaks.

**Figure 10:**
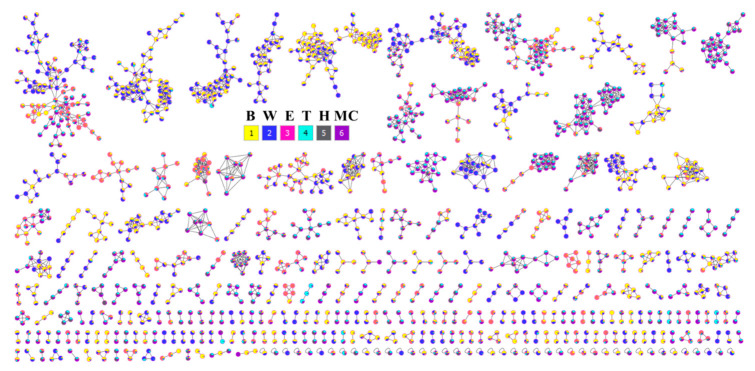
Feature-based molecular network of extract (Neon Blue) and fractions [H: Black; MC: Purple; E: Magenta; B: Yellow; W: Blue] of leaves (GNPS molecular network with job ID = cfa6443e13824edf8132a3f391ad91f3) of *I*. *rotunda*.

Nodes in the molecular network were generated using the GNPS platform and visualized. Precursors were grouped into clusters by collecting nodes that showed similar isotope patterns (Figure 10).

All samples were subjected to high-resolution tandem mass spectrometry using data-dependent acquisition experiments in collaboration with open-source web platforms, which enabled access to public mass libraries. Chemical annotation involved obtaining the retention time, precursors, and characteristic fragmentation with web tools from online mass database tools. In total, 128 compounds from twigs and 89 compounds from leaves (Tables S1 and S2, Supplementary Materials) of *I*. *rotunda* were putatively identified and dereplicated with mass errors of less than 5 mDa through an integrated strategy. The twig extract revealed abundant components, including 33 phenolics, 32 terpenes, 25 fatty acids, 11 organic acids, 9 glycosides, 4 alkaloids, 1 iridoid, and 1 glycerol, along with 12 undescribed structures. Moreover, the leaf extract primarily consisted of 20 phenolics, 17 terpenes, 18 fatty acids, 11 organic acids, 6 glycosides, 1 alkaloid, 3 flavonoids, and 13 undescribed structures.

## 3. Discussion

*I*. *rotunda* is a famous folk medicine that has been extensively utilized and developed for detoxification and relieving pain. In this study, we newly established a green technique to prepare leaf and twig extracts of this plant, aiming to discover its ethnopharmacological application for treating inflammation. The extracts from the leaves and twigs of *I*. *rotunda* were successfully extracted and partitioned into fractions. To elucidate the anti-inflammatory effects of this plant in traditional medicine, we investigated the anti-inflammatory potential of *I*. *rotunda* by accessing bioactivities of its extracts and fractions using LPS-stimulated RAW264.7 macrophages. Inflammatory diseases are significantly influenced by nitrite oxide and pro-inflammatory cytokines generated by activated macrophages [21]. Thus, modulating NO and pro-inflammatory cytokine production in inflammatory cells represents a therapeutic strategy for managing diseases inflammatory associated with inflammation [10]. The results demonstrated that extracts and fractions from leaves and twigs of *I*. *rotunda* significantly decreased expression levels of NO production in LPS-stimulated RAW264.7 cells. Notably, all leaf fractions except the W fraction potently inhibited NO production, exhibiting greater potency than positive control. These findings suggested that the active ingredients responsible for the inhibitory effects of *I*. *rotunda* are predominantly found in the H, MC, EA, and B fractions. Notably, these active fractions did not impact the viability of RAW264.7 cells at concentrations up to 30 µg/mL, indicating that these fractions specifically target inflammatory processes. Similarly, the extract and H, MC, E, and B fractions from twigs of *I*. *rotunda* markedly downregulated LPS-induced NO production in RAW264.7 cells without showing cytotoxicity at concentrations up to 20 µg/mL (MC fraction) and 30 µg/mL (all twig fractions), indicating that these active fractions could contain active ingredients with anti-inflammatory effects.

NO is produced during the conversion of L-arginine into L-citrulline catalyzed by iNOS. Excessive generation of NO has been associated with septic shock, rheumatoid arthritis, and inflammatory conditions [22]. iNOS plays critical roles in these inflammatory conditions [23]. To assess the molecular actions of active fractions from leaf and twig extracts of *I*. *rotunda* on RAW264.7 cells, two extracts and their active fractions were examined for their regulatory effects on iNOS and COX-2 protein expression. Among leaf samples, H and E fractions dramatically suppressed COX-2 expression levels. Additionally, H, MC, and E fractions significantly reduced iNOS expression levels. Notably, the inhibitory effects of extracts and fractions from *I*. *rotunda* leaves on the LPS-stimulated expression of iNOS and COX-2 in RAW264.7 cells were not due to their cytotoxicity, as assessed by comparing their expression levels against the housekeeping gene β-actin. Similarly, the extracts and H and E fractions from twigs strongly reduced the iNOS expression level without having any effect on RAW264.7 cell viability at the tested concentration. On the other hand, extracts and fractions (H and E) from twigs significantly mediated COX-2 expression without showing any cytotoxicity to LPS-stimulated RAW264.7 cells. Among them, the MC fractions from twigs of *I*. *rotunda* demonstrated toxic effects on LPS-stimulated RAW264.7 cells. The findings indicated that active extracts and fractions from leaves and twigs of *I*. *rotunda* without toxic effects suppressed NO and PGE2 production through active ingredients that downregulated iNOS and COX-2 expression.

Previous studies have demonstrated that the reduction in iNOS expression in macrophages is affected by TNF-α alteration [24,25]. TNF-α production is crucial for the synergistic induction of NO synthesis in IFN-γ- and/or LPS-stimulated macrophages. Consequently, the extracts and fractions from leaves and twigs of *I*. *rotunda* were assessed for their effects on LPS-induced TNF-α production in RAW264.7 cells. H and MC fractions, along with the leaf extract, dramatically inhibited TNF-α production without affecting cell viability. The extracts and MC fraction of twigs also significantly suppressed TNF-α production without showing cytotoxicity. Moreover, the extract and H and MC fractions from leaves potentially inhibited IL-6 production without affecting cell viability at concentrations up to 30 µg/mL. The MC fraction of twigs also suppressed LPS-induced IL-6 production at a concentration of 10 µg/mL without showing any cytotoxicity based on the cell viability assay. This study suggested that the extracts and active fractions of the leaves and twigs of *I*. *rotunda* could dose-dependently reduce the expression or production of TNF-α and IL-6 in activated RAW264.7 macrophages, therefore alleviating LPS-induced cellular inflammatory responses. Indeed, these active samples suppressed LPS-induced TNF-α and IL-6 production in RAW264.7 cells through the active ingredients they contained.

NFκB can influence inflammation responses by modulating the release of IL-6, TNF-α, and anti-inflammatory enzymes (iNOS, COX-2) [26]. To investigate the mechanism of inhibitory effects in the ELISA assay, these active fractions were further studied by using Western blotting analysis. The leaf extract and active (H and MC) fractions notably inhibited the phosphorylation of p65 by reducing phosphorylation of IκBα and downregulating the degradation of IκBα. Similarly, the twig extract and its MC fraction significantly decreased the phosphorylation of p65 by inhibiting the phosphorylation of IκBα, higher than effects of H and E fractions. Therefore, these extracts and fractions from the leaves and twigs of *I*. *rotunda* might inhibit NO, IL-6, TNF-α production, and iNOS expression level by blocking NFkB action through inhibiting p65 phosphorylation and IκBα degradation. Thus, the extracts and active fractions of leaves of *I*. *rotunda* could be useful for downregulating inflammatory responses.

These active extracts and fractions also suppressed the phosphorylation of p38 and ERK, effectively inhibiting the production of IL-6 and TNF-α and COX-2 expression levels, thereby exerting an anti-inflammatory action [27]. The extract and fractions (H and MC) showed an ability to reduce the LPS-induced phosphorylation of ERK in RAW264.7 cells without having any toxic effects on β-actin, a housekeeping gene, suggesting that these samples suppressed the ERK MAPK signaling pathway through active constituents in the extract and fractions from leaves of *I*. *rotunda*. Accordingly, the extract and H and MC fractions may inhibit the production of IL-6 and TNF-α through the ERK MAPK pathway [28]. Additionally, the extract and MC fraction of twig also downregulated the phosphorylation of ERK in LPS-activated macrophages without having any toxic effects on β-actin. The E fraction from twig also inhibited the phosphorylation of p38 MAPK induced by LPS-activated macrophages without affecting β-actin. This observation indicates that the extracts and active fractions from twigs of *I*. *rotunda* may inhibit IL-6 and TNF-α production by deactivating MAPK through either the p38 or ERK signaling pathways, mediated by active ingredients from the *I*. *rotunda* twigs. Extracts and fractions from leaves and twigs of *I*. *rotunda* were also evaluated for their antioxidative activities based on their ability to scavenge DPPH and ABTS radicals. However, they did not show much antioxidative effects compared to positive control.

LC-MS/MS is widely used in the analysis of herbal medicine due to its high accuracy and sensitivity. Specially, non-target metabolomics approaches profiling the metabolome can provide comprehensive information on metabolites [29]. This technique enables the analysis of mass isotope patterns of detected signals and comparison with those reported from public mass database libraries [30]. In this study, a non-target strategy was applied to analyze and provide a comprehensive identification of 217 compounds from extracts and fractions of leaves and twigs of *I*. *rotunda*. In contrast, twig extracts and fractions demonstrated more nodes clustered into a greater number of consensus clusters (Figure 9 and Figure 10). Their potent anti-inflammatory effects may stem from the high intensity and abundance of peak compositions in the MC fractions of both leaves and twigs of this plant. Hence, it was proposed that these active fractions be further isolated to identify active compounds with anti-inflammatory effects. When major components were compared, extracts and fractions of leaves and twigs indicated that the phenolic class was the largest group of differential metabolites. In particular, the phenolic content of the twig organ was 26%, exceeding the 22% found in leaf extract (Figures S2–S4, Supplementary Materials). Phenolics are prominent secondary metabolites in plants, playing an important role throughout the entire metabolic pathway of the natural biosynthesis of metabolites [31]. The twig organ also showed that the high content of terpene group was about 25% compared to 20% in the leaf organ. Terpenes are known as bioactive compounds with vital roles in human health and potential applications in developing natural anti-inflammatory [32], anti-diabetic, and anti-microbial products [33].

Phenolics are considered to have health benefits due to their association with various biological effects such as anti-inflammatory [34], antioxidative, and antifungal [35] activities. Indeed, the antioxidative and anti-inflammatory activities of leaf and twig extracts of *I*. *rotunda* correlate with their phenolic content. The twig extract showed a higher antioxidative and anti-inflammatory potential than the leaf extract. Notably, the MC fraction from twigs displayed a higher content of organic acids than other fractions from twigs, correlating with results showing that it exhibited stronger DPPH radical scavenging activity and inhibitory effects against TNF-α, IL-6, iNOS, COX-2, pERK, and p65 expression levels. A previous study indicated that organic acids significantly contribute to counteracting the effects of oxidants and inflammation [36]. The leaf extract and its fractions showed similar compound group compositions except for flavonoids, which were identified from all leaf samples. Noteworthily, the B and W fractions from the leaf extract displayed lower contents of organic acids than the H, MC, and EA fractions, reflecting their varying antioxidative and anti-inflammatory activities.

## 4. Materials and Methods

### 4.1. Plant Materials

Twigs and leaves of *I*. *rotunda* were collected from Sunchon National University (Gurye, Republic of Korea) in November 2023 and identified by Professor Mina Lee (College of Pharmacy, Sunchon National University). A voucher specimen (SCNUP 27-2024) is preserved at the Pharmacognosy Laboratory, College of Pharmacy, Sunchon National University, Suncheon-si, Jeonnam-do, Korea.

### 4.2. Extraction and Fractionation Conditions

Leaf and twig samples were pretreated and extracted using a supercritical fluid extraction system, following the same procedures: the temperature was set at 50 °C before injection into the system. Once the equipment’s temperature stabilized, the sample was introduced via a high-pressure pump, followed by injecting CO_2_ until the pressure reached 400 bar. The valve was adjusted to ensure that the pressure inside the equipment changed linearly over time and was maintained at a constant level. Ethanol (300 mL) was added as a co-solvent at a rate of 5 mL for 120 min. The resulting solution was then concentrated under vacuum to obtain residue (200 g). This residue was subsequently suspended in distilled water, then partitioned with solvents of increasing polarity from *n*-hexane to *n*-BuOH to obtain H, MC, E, B, and W, respectively. These extracts and fractions were stored at 4 °C in the refrigerator for further use.

### 4.3. Biological Assays

#### 4.3.1. Determination of Anti-Inflammatory Mediator and Cytokines

##### Cell Culture and Viability

RAW264.7 cells were maintained in Dulbecco’s modified Eagle’s medium supplemented with 10% heat-inactivated FBS, penicillin (100 IU/mL), and streptomycin sulfate (100 µg/mL). The cells were cultured under normoxic conditions in a humidified atmosphere at 37 °C with 5% CO_2_. After 24 h, cells were implemented with extracts and fractions. In brief, cells were stimulated with 1 µg/mL LPS, followed by incubation for 16 h in 96-well plates.

##### Measurement of NO Production

After pretreatment for 2 h with *Ilex rotunda*’s candidates, RAW264.7 cells were stimulated with LPS (100 ng/mL) for 24 h. On the next day, the cell culture supernatant was collected and transferred into a 96-well plate containing 100 µL/well. The level of NO production was determined by mixing with 1% (*w/v*) sulfanilamide in 5% (*v/v*) phosphoric acid and 0.1% (*w/v*) N-(1-naphthyl) ethylenediamine dihydrochloride and incubation at room temperature for 10 min. The absorbance was measured at 550 nm using microplate reader (Bio-Tek Instruments, Inc., Winooski, VT, USA) [37].

##### ELISA Assay

Interleukin-6 (IL-6) and tumor necrosis factor alpha (TNF-α) were assessed using an ELISA kit (BD OptEIATM, San Diego, CA, USA) following the manufacturer’s instructions. The RAW264.7 cell line was utilized to determine the synthesis of inflammatory factors, with cells seeded at 5 × 10^4^ cells/well in 96 -well plates. RAW264.7 cells were pre-incubated with or without the candidates at designated concentrations for 2 h before stimulation with LPS for 24 h. IL-6 and TNF-α levels were assessed by collecting the cell culture media post stimulation.

#### 4.3.2. Western Blot Assay

RAW264.7 cell lines (purchased from the Korean Cell Line Bank, Seoul, Republic of Korea) were used as a testing model for Ilex rotunda’s candidates. After pretreatment for 2 h, the cells were stimulated with LPS (Lipopolysaccharide 100 ng/mL) for specified duration and then lysed with PRO-PREPTM (Intron Biotechnology, Seoul, Republic of Korea) supplemented using the Pierce™ Bradford Protein Assay Kit (Thermo Fisher Scientific. (n.d.). Pierce™ Bradford Protein Assay Kit, Waltham, MA, USA). Proteins (20 μg) were separated by SDS-PAGE using 8% and 10% acrylamide gels and transferred to a PVDF membrane. Antibodies including P-p44/42 MAPK (#9101), p44/42 (#4695), P-p38 (#9211), p38 (#9212), p-IKKα/β (#2967), p-IκBα (#9246), p-p65 (#3033), p65 (#8242), IKKα (#2682), and IKKβ (#2370) were procured from Cell Signaling Technology (Danvers, MA, USA). IκBα (SC-371) antibodies were obtained from Santa Curz Biotechnology Inc., (Santa Cruz, CA, USA). β-actin (AB_2289199), iNOS (AB_397808), and COX-2 (AB_397603) were purchased from BD Bioscience (San Jose, CA, USA)10. After being transferred to PVDF membranes, they were blocked for 2 h with 5% skim milk before incubation with the primary antibodies diluted 1:1000 in 2.5% skim milk and incubated at 4 °C for 18 h, and the blots were washed three times with Tween20/Tris-buffered saline (T/TBS) and incubated with an HRP-conjugated secondary antibody diluted 1:2000 in 5% skim milk for 2 h at room temperature, followed by three additional washes with T/TBS. The expression of the targeted protein was detected using Super Signal ™ West Femto Maximum Sensitivity Substrate (Thermo Fisher Scientific, Waltham, MA, USA).

### 4.4. LC-MS/MS Data Analysis

#### 4.4.1. LC-MS/MS Conditions

The LC-MS/MS system included a Vanquish UHPLC system decoupled with an Orbitrap Exploris 120 mass spectrometer (Thermo Fisher Scientific, Sunnyvale, CA, USA). The chromatographic separation was performed using a Waters Acquity UPLC HSS T3 column (4.6 × 100 mm, 1.8 μm, Waters, Milford, MA, USA) at 40 °C with a flow rate of 0.3 mL/min and an injection volume of 4 μL. A gradient solvent system consisted of phase A (pure water containing 0.1% formic acid) and phase B (ACN, 0.1% formic acid), applied as follows: 8–15% (B) for 0–4 min, 15–32% (B) for 4–8 min, 32–53% (B) for 8–11 min, 53–100% (B) for 11–24 min and held for three minutes, and 100–8% (B) for 1 min before re-equilibrium with 8% (B). The sample solutions were filtered using a 0.22 μm PTFE membrane (Agilent Technologies, Santa Clara, CA, USA) prior to further analysis.

#### 4.4.2. Molecular Networking

The molecular network was constructed on the Global Natural Products Social (GNPS) web platform (https://gnps.ucsd.edu, accessed on 19 September 2024), facilitating the chemical networking of extracts and fractions from leaves and twigs of *I*. *rotunda*. Initially, the output file from UHPLC-MS/MS analysis was transferred into mzXML format by using MS converter tool (https://proteowizard.sourceforge.io/, accessed on 10 September 2024). Then, analysis performed on the GNPS web platform yielded a molecular network (twig job ID: ID = 96ad1b5bbed84c8bb073d8c882f3897b1; leaf job ID = cfa6443e13824edf8132a3f391ad91f3), with each node correlating to a unique MS^2^ spectrum or a consensus cluster of identical MS^2^ spectra. Cosine scores, reflecting spectral similarity based on precursor ions, fragment ions, and peak intensities, were computed between distinct but related MS^2^ spectra nodes established using cosine similarity scores. Data visualization and network annotation propagation (NAP) were conducted by using the Cytoscape 3.9.0 program (https://cytoscape.org/, accessed on 25 September 2024).

#### 4.4.3. LC-MS Annotations

Component identification from extracts and fractions was performed using open-source tools such as GNPS web-tools and MS-Dial version 5.1 coupled with public mass databanks (GNPS, HMDB, Lipidmaps, KNApSAcK, American mass bank). The compound annotations involved analyzing and comparing precursor and fragmentation ions against those in public MS/MS databases for spectral matching.

#### 4.4.4. Statistical Analysis

Data were expressed as the mean ± standard error of the mean (*n* = 3). Statistically significant values were analyzed using one-way analysis of variance (ANOVA) and Dunnett’s post hoc test with GraphPad Prism software version 8.0 (GraphPad Software, Inc., San Diego, CA, USA). Differences were considered significant at * *p* < 0.05 and ** *p* < 0.001 compared to the controls.

## 5. Conclusions

A green extraction method was successfully applied to extract *I*. *rotunda*, resulting in the partition of five fractions from the leaf or twig extract, respectively, for the first time. Pretreatment with the extract and active fractions of leaves and twigs of *I*. *rotunda* reduced NO production and expression levels of pro-inflammatory genes, including those encoding IL-6, TNF-α, iNOS, and COX-2 in LPS-activated RAW 264.7 macrophages by suppressing the phosphorylation of NFkB p65, IκBα, p38 MAPK, and ERK MAPK. Therefore, these active extracts and fractions from leaves and twigs of *I*. *rotunda* could potentially be developed into therapeutic products to treat or prevent inflammatory diseases. This study could serve as a foundation and guide for future research identifying active components in extracts or fractions using a non-targeted metabolomics approach, saving time and effort in discovering compounds with anti-inflammatory activities. Further studies are needed to separate active components and evaluate their bioactivities targeting inflammation.

## Data Availability

The original contributions presented in this study are included in this article/Supplementary Materials; further inquiries can be directed to the corresponding author/s.

## Supplementary Materials

The following supporting information can be downloaded at https://www.mdpi.com/article/10.3390/ijms252211965/s1.
