# Supplementary material for: Anti-Inflammatory Effects and Metabolomic Analysis of Ilex Rotunda Extracted by Supercritical Fluid Extraction
Source: Int J Mol Sci. 2024 Nov 7;25(22):11965. doi: 10.3390/ijms252211965 (PMC11593382; doi:10.3390/ijms252211965)
Supplement: Supplementary file 1 [file ijms-25-11965-s001.zip › ijms-3286558-supplementary.pdf]

Supplementary materials

Anti-inflammatory effects and metabolomic analysis of *Ilex rotunda* extracted by supercritical fluid extraction

Duc Dat Le [1,2,†], Young Su Jang[2,†], Vinhquang Truong [1], Thientam Dinh [1], Thinhulinh Dang [1], Soojung Yu [3], and Mina Lee [1,3,*]

[1] College of Pharmacy and Research Institute of Life and Pharmaceutical Sciences, Sunchon National University, 255 Jungangno, Suncheon 57922, Jeonnam, Republic of Korea; D.D.L (ddle@scnu.ac.kr); V.T (quangvtruong00@gmail.com); T.T (thientamm.2001@gmail.com)

[2] Nano Bio Research Center, Jeonnam Bio Foundation, Jangseong 57248, Jeonnam, Republic of Korea; Y.S.J (ysjang@jbf.kr)

[3] Department of Natural Cosmetics Science, Graduate School, Sunchon National University, 255 Jungangno, Suncheon 57922, Jeonnam, Republic of Korea; S.Y (ysj1997s@naver.com)

[†] These authors contributed equally to this work.

*Correspondence: minalee@sunchon.ac.kr/ minalee@scnu.ac.kr (M. L); Tel.: +82-61-750-3764; Fax: +82-61-750-3708

Table S1. Identification of components from total, H, MC, E, B, and W extracts of leaves of *Ilex rotunda*.

No.	Compound	RT (min)	Total	H	MC	EA	Bu	DW	Formula	Adduct	*m/z* (Da)	Error (mDa)	Class
1	Sorbitol[#]	0.803	+	+	+	+	+	+	$C_6H_{14}O_6$	$[M-H]^-$	181.0718	1.780	Glycoside
2	L-sorbose[#]	0.807	+	+	+	+	+	+	$C_6H_{12}O_6$	$[M-H]^-$	179.0562	0.198	Glycoside
3	Xylitol[#]	0.809	+	+	+	+	+	+	$C_5H_{12}O_5$	$[M-H]^-$	151.0612	0.198	Glycoside
4	Sucrose[$]	0.809	+	+	+	+	+	+	$C_{12}H_{22}O_{11}$	$[M-H]^-$	341.1088	0.214	Glycoside
5	Quinic acid[#]	1.493	+	+	+	+	+	+	$C_7H_{12}O_6$	$[M-H]^-$	191.0562	0.137	Organic acid
6	Acetosyringone[#]	1.556	+	+	+	+	+	+	$C_{10}H_{12}O_4$	$[M-H]^-$	195.0663	0.305	Phenolic
7	Succinic acid[#]	1.159	+	-	-	-	+	+	$C_4H_6O_4$	$[M-H]^-$	177.0193	0.031	Organic acid
8	Unknown1[#]	1.193	+	-	-	+	+	+	$C_{11}H_{20}O_7$	$[M-H]^-$	263.1136	-	Unknown
9	2-Oxoadipic acid[#]	2.413	+	+	+	+	+	+	$C_6H_8O_5$	$[M-H]^-$	159.0299	0.061	Organic acid
10	Vanillin[#]	3.440	+	+	+	+	+	+	$C_8H_8O_3$	$[M-H]^-$	151.0401	1.114	Phenolic
11	Unknown2[#]	3.522	+	+	+	+	+	+	$C_{10}H_{16}O_6$	$[M-H]^-$	231.0874	-	Unknown
12	Unknown3[#]	3.9	+	+	+	+	+	+	$C_{10}H_{16}O_6$	$[M-H]^-$	231.0876	-	Unknown
13	Unknown4[#]	3.975	+	+	+	+	+	+	$C_{10}H_{12}O_8N_6$	$[M-H]^-$	343.0645	-	Unknown
14	3-Hydroxybenzoic acid[#]	4.089	+	+	+	+	+	+	$C_7H_6O_3$	$[M-H]^-$	137.0244	0.001	Phenolic
15	6-Carboxyhexanoate[#]	4.773	+	+	+	+	+	+	$C_7H_{12}O_4$	$[M-H]^-$	159.0664	0.244	Fatty acid
16	Vanillic acid[†]	4.911	+	+	+	+	+	+	$C_8H_8O_4$	$[M-H]^-$	167.035	0.977	Phenolic
17	Unknown6[#]	5.016	+	+	+	+	+	+	$C_{18}H_{26}N_4O_6$	$[M-H]^-$	395.192	-	Unknown
18	Unknown7[#]	5.078	+	+	+	+	+	+	$C_{12}H_{20}O_9$	$[M-H]^-$	307.1037	-	Unknown
19	Syringic acid[#]	5.286	+	+	+	+	+	+	$C_9H_{10}O_5$	$[M-H]^-$	197.0456	0.041	Phenolic

Table S1. Cont.

No.	Compound	RT (min)	Total	H	MC	EA	Bu	DW	Fomula	Adduct	*m/z* (Da)	Error (mDa)	Class
20	6,7-Dihydroxycoumarin#	5.353	+	+	+	+	+	+	$C_9H_6O_4$	$[M-H]^-$	177.0193	0.030	Phenolic
21	Unknown9#	5.476	+	+	+	+	+	+	$C_{12}H_{20}O_9$	$[M-H]^-$	307.1037	-	Unknown
22	Osmanthuside H#	5.490	+	+	+	+	+	+	$C_{19}H_{28}O_{11}$	$[M-H]^-$	431.192	3.598	Glycoside
23	Unknown10#	5.615	+	+	+	+	+	+	$C_{10}H_{14}O_7$	$[M-H]^-$	245.0668	-	Unknown
24	2-methylidene-4-[(2R,3R,4S,5S,6R)-3,4,5-trihydroxy-6-(hydroxymethyl)oxan-2-yl]oxybutanoic acid#	5.615	+	+	+	+	+	+	$C_{11}H_{18}O_8$	$[M-H]^-$	277.093	0.000	Glucosides
25	Benzoic acid#	5.705	+	+	+	+	+	+	$C_7H_6O_2$	$[M-H]^-$	121.0294	0.015	Organic acid
26	4,6,8-trihydroxy-7-methoxy-3-methyl-3,4-dihydroisochromen-1-one#	6.508	+	+	+	+	+	+	$C_{11}H_{12}O_6$	$[M-H]^-$	239.0562	0.061	Organic acid
27	*Trans*-*p*-hydroxycinnamic acid#	6.889	+	+	+	+	+	+	$C_9H_8O_3$	$[M-H]^-$	163.0401	0.061	Phenolic
28	Esculetin#	6.893	+	+	+	+	+	+	$C_9H_6O_4$	$[M-H]^-$	177.0193	0.030	Phenolic
29	Tropic acid#	7.069	+	+	+	+	+	+	$C_9H_{10}O_3$	$[M-H]^-$	165.0558	0.031	Organic acid
30	Umbelliferone#	7.191	+	+	+	+	+	+	$C_9H_6O_3$	$[M-H]^-$	161.0244	0.039	Phenolic
31	Isoscopoletin#	7.415	+	+	+	+	+	+	$C_{10}H_8O_4$	$[M-H]^-$	191.035	0	Phenolic
32	Sinapic acid#	7.434	+	+	+	+	+	+	$C_{11}H_{12}O_5$	$[M-H]^-$	223.0613	0.028	Phenolic
33	Kaempferol-3-*O*-rutinoside#	7.767	+	+	+	+	+	+	$C_{27}H_{30}O_{15}$	$[M-H]^-$	593.1509	4.913	Flavonoid
34	9-(2,3-dihydroxypropoxy)-9-oxononanoic acid#	7.782	+	+	+	+	+	+	$C_{12}H_{22}O_6$	$[M-H]^-$	261.1344	0.039	Fatty acid
35	(-)-Pinoresinol glucoside#	7.930	+	+	+	+	+	+	$C_{36}H_{32}O_{11}$	$[M-H]^-$	519.1869	0.012	Phenolic
36	Unknown16#	7.977	+	+	+	+	+	+	$C_{11}H_{18}O_4$	$[M-H]^-$	213.1132	-	Unknown
37	Nephromopsic acid#	8.122	+	-	+	-	-	+	$C_{19}H_{34}O_4$	$[M-H]^-$	325.2275	0.183	Organic acid

Table S1. Cont.

No.	Compound	RT (min)	Total	H	MC	EA	Bu	DW	Formula	Adduct	*m/z* (Da)	Error (mDa)	Class
38	(+)-Syringaresinol *β*-D-glucoside #	8.050	+	+	+	+	+	+	$C_{28}H_{36}O_{13}$	$[M-H]^-$	579.2081	0.085	Phenolic
39	Coniferylaldehyde#	8.443	+	+	+	+	+	+	$C_{10}H_{10}O_3$	$[M-H]^-$	177.0556	0.732	Phenolic
40	Sinapoyl aldehyde#	8.483	+	+	+	+	+	+	$C_{11}H_{12}O_4$	$[M-H]^-$	207.0663	0.030	Phenolic
41	Azelaic acid#	8.483	+	+	+	+	+	+	$C_9H_{16}O_4$	$[M-H]^-$	187.0976	0.041	Organic acid
42	3-Hydroxybenzoate#	8.577	+	+	+	+	+	+	$C_7H_6O_3$	$[M-H]^-$	137.0245	0.001	Organic acid
43	FA 9:0+1O#	8.720	+	+	+	+	+	+	$C_9H_{18}O_3$	$[M-H]^-$	173.1183	0.076	Phenolic
44	Dihydrokaempferol#	8.756	+	+	+	+	+	+	$C_{15}H_{12}O_6$	$[M-H]^-$	287.0561	0.269	Flavonoid
45	6-Hydroxynicotinate#	8.891	+	+	+	+	+	+	$C_6H_5NO_3$	$[M-H]^-$	138.0196	0.031	Alkaloid
46	(2R,3R,4R,6aR,6bS,8aS,11R,12R,12aS,14bR)-2,3,12-trihydroxy-4,6a,6b,11,12,14b-hexamethyl-8a-[(2S,3R,4S,5S,6R)-3,4,5-trihydroxy-6-(hydroxymethyl)oxan-2-yl]oxycarbonyl-1,2,3,4a,5,6,7,8,9,10,11,12a,14,14a-tetradecahydropicene-4-carboxylic acid#	9.193	+	+	+	+	+	+	$C_{36}H_{56}O_{12}$	$[M-H]^-$	679.3698	0.122	Fatty acid
47	Methyl (4S,5Z,6S)-5-(2-acetyloxyethylidene)-4-[2-[2-(4-hydroxyphenyl)ethoxy]-2-oxoethyl]-6-[(2S,3R,4S,5S,6R)-3,4,5-trihydroxy-6-(hydroxymethyl)oxan-2-yl]oxy-4H-pyran-3-carboxylate#	9.563	+	+	+	+	+	+	$C_{27}H_{34}O_{14}$	$[M-H]^-$	581.1873	0.067	Phenolic
48	Abscisic acid#	9.750	+	+	+	+	+	+	$C_{15}H_{20}O_4$	$[M-H]^-$	263.1289	0.091	Organic acid
49	(*E*)-3,4,5-Trimethoxycinnamic acid#	9.944	+	+	+	+	+	+	$C_{12}H_{14}O_5$	$[M-H]^-$	237.0769	0.027	Phenolic
50	Syringaresinol§	10.070	+	+	+	+	+	+	$C_{22}H_{26}O_8$	$[M-H]^-$	417.1551	0.091	Phenolic
51	Naringenin#	10.774	+	+	+	+	+	+	$C_{15}H_{12}O_5$	$[M-H]^-$	271.061	0.030	Flavonoid
52	(*E*,9*S*)-11-[(1*S*,2*R*,3*R*)-2-ethyl-3-hydroxy-5-oxocyclopentyl]-9-hydroxyundec-10-enoic acid#	10.788	+	+	+	+	+	+	$C_{18}H_{30}O_5$	$[M-H]^-$	325.2021	0.091	Fatty acid
53	(10*E*,15*E*)-9,12,13-trihydroxyoctadeca-10,15-dienoic acid#	10.826	+	+	+	+	+	+	$C_{18}H_{32}O_5$	$[M-H]^-$	327.2177	0.027	Fatty acid

Table S1. Cont.

No.	Compound	RT (min)	Total	H	MC	EA	Bu	DW	Formula	Adduct	*m/z* (Da)	Error (mDa)	Class
54	Acaranoic acid#	11.111	+	+	+	+	+	+	$C_{18}H_{36}O_5$	$[M-H]^-$	331.2491	0.0867	Fatty acid
55	(3*S*,4*R*,6a*R*,6b*S*,8a*S*,11*R*,12*R*,14b*R*)-3,12-dihydroxy-4,6a,6b,11,12,14b-hexamethyl-8a-[3,4,5-trihydroxy-6-(hydroxymethyl)oxan-2-yl]oxycarbonyl-1,2,3,4a,5,6,7,8,9,10,11,12a,14,14a-tetradecahydropicene-4-carboxylic acid#	10.842	+	+	+	+	+	+	$C_{36}H_{56}O_{11}$	$[M-H]^-$	663.3749	0.012	Terpene
56	BRD-A33294632-001-01-1#	10.933	+	+	+	+	+	+	$C_{36}H_{58}O_{10}$	$[M+HCOO]^-$	695.4011	0.012	Terpene
57	(Z)-9,12,13-trihydroxyoctadec-15-enoic acid#	11.243	+	+	+	+	+	+	$C_{18}H_{34}O_5$	$[M-H]^-$	329.2334	0.275	Fatty acid
58	(3*S*,4*R*,6a*R*,6b*S*,8a*S*,11*R*,12*R*,14b*R*)-3,12-dihydroxy-4,6a,6b,11,12,14b-hexamethyl-8a-[3,4,5-trihydroxy-6-(hydroxymethyl)oxan-2-yl]oxycarbonyl-1,2,3,4a,5,6,7,8,9,10,11,12a,14,14a-tetradecahydropicene-4-carboxylic acid#	11.819	+	+	+	+	+	+	$C_{36}H_{56}O_{11}$	$[M-H]^-$	663.375	0	Terpene
59	(2*S*,3*S*,4*S*,5*R*,6*R*)-6-[[(3*S*,6a*R*,6b*S*,8a*S*,14b*R*)-4,4,6a,6b,11,11,14b-heptamethyl-8a-[(2*S*,3*R*,4*S*,5*S*,6*R*)-3,4,5-trihydroxy-6-(hydroxymethyl)oxan-2-yl]oxycarbonyl-1,2,3,4a,5,6,7,8,9,10,12,12a,14,14a-tetradecahydropicen-3-yl]oxy]-3,4,5-trihydroxyoxane-2-carboxylic acid#	12.486	+	+	+	+	+	+	$C_{42}H_{66}O_{14}$	$[M-H]^-$	793.4382	0.018	Terpene
60	Medicagenic acid#	12.865	+	+	+	+	+	+	$C_{30}H_{46}O_6$	$[M-H]^-$	501.3219	2.350	Terpene
61	6-Gingerol#	13.050	+	+	+	+	+	+	$C_{17}H_{26}O_4$	$[M-H]^-$	293.1759	0.091	Phenolic
62	Asiatic acid#	13.318	+	+	+	+	+	+	$C_{30}H_{48}O_5$	$[M-H]^-$	487.3427	0.244	Terpene
63	13-Keto-9Z,11E-octadecadienoic acid#	13.385	+	+	+	+	+	+	$C_{18}H_{30}O_3$	$[M-H]^-$	293.2123	1.282	Fatty acid
64	(1R,2S,5aR,5bR,7aS,10R,12bR)-2-Hydroxy-10-isopropenyl-3,3,5a,5b,12b-pentamethyloctadecahydrodicyclopenta[a,i]phenanthrene-1,7a(1H)-dicarboxylic acid#	13.906	+	+	+	+	+	+	$C_{30}H_{46}O_5$	$[M-H]^-$	485.3267	0.018	Terpene
65	Unknown28#	14.656	+	+	+	+	+	+	$C_{29}H_{44}O_5$	$[M-H]^-$	471.311	-	Unknown
66	9,12,15-Octadecatrienoic acid, 3-(hexopyranosyloxy)-2-hydroxypropyl ester, (9Z,12Z,15Z) #	15.130	+	+	+	+	+	+	$C_{27}H_{46}O_9$	$[M-H]^-$	559.3121	0.061	Fatty acid

Table S1. Cont.

No.	Compound	RT (min)	Total	H	MC	EA	Bu	DW	Formula	Adduct	*m/z* (Da)	Error (mDa)	Class
67	(9S,10E,12Z,15Z)-9-Hydroxy-10,12,15-octadecatrienoic acid[#]	15.161	+	+	+	+	+	+	$C_{18}H_{30}O_3$	$[M-H]^-$	293.2124	0.003	Fatty acid
68	13-HOTE[#]	15.346	+	+	+	+	+	+	$C_{18}H_{30}O_3$	$[M-H]^-$	293.2123	0.003	Fatty acid
69	Unknown29[#]	15.554	+	+	+	+	+	+	$C_{29}H_{44}O_4$	$[M-H]^-$	455.3163	-	Unknown
70	MEHP[#]	15.814	+	+	+	+	+	+	$C_{16}H_{22}O_4$	$[M-H]^-$	277.1446	0.039	Organic acid
71	Maslinic acid[†]	15.986	+	+	+	+	+	+	$C_{30}H_{48}O_4$	$[M-H]^-$	471.3476	0.035	Terpene
72	*α*-Dimorphecolic acid[#]	16.261	+	+	+	+	+	+	$C_{18}H_{32}O_3$	$[M-H]^-$	295.228	0.003	Fatty acid
73	Unknown30[#]	16.473	+	+	+	+	+	+	$C_{39}H_{52}O_6$	$[M-H]^-$	617.3846	-	Unknown
74	*Trans*-3-feruloylcorosolic acid[#]	16.773	+	+	+	+	+	+	$C_{40}H_{56}O_7$	$[M-H]^-$	647.3951	0.002	Terpene
75	*Cis*-p-coumaroylcorosolic acid[#]	16.944	+	+	+	+	+	+	$C_{39}H_{54}O_6$	$[M-H]^-$	617.3847	0.001	Terpene
76	Norselic acid D[#]	17.098	+	+	+	+	+	+	$C_{29}H_{44}O_4$	$[M-H]^-$	455.3164	0.002	Terpene
77	Ricinoleic acid[#]	17.295	+	+	+	+	+	+	$C_{18}H_{34}O_3$	$[M-H]^-$	297.2435	0.004	Fatty acid
78	13*S*-hydroxyoctadecadienoic acid[#]	17.642	+	+	+	+	+	+	$C_{18}H_{32}O_3$	$[M-H]^-$	295.2278	0	Fatty acid
79	Hederagenin[#]	18.197	+	+	+	+	+	+	$C_{30}H_{48}O_4$	$[M-H]^-$	471.3474	0.003	Terpene
80	Camelledionol[#]	19.367	+	+	+	+	+	+	$C_{29}H_{44}O_3$	$[M-H]^-$	439.3222	0.002	Terpene
81	Linolenic acid[#]	20.041	+	+	+	+	+	+	$C_{18}H_{30}O_2$	$[M-H]^-$	277.2173	0.006	Fatty acid
82	Oleanolic acid[§, $]	20.336	+	+	+	+	+	+	$C_{30}H_{48}O_3$	$[M-H]^-$	455.3528	0.005	Terpene
83	Ursolic acid[§, †, $]	20.698	+	+	+	+	+	+	$C_{30}H_{48}O_3$	$[M-H]^-$	455.3528	0.002	Terpene
84	Betulinic acid[#]	21.009	+	+	+	+	+	+	$C_{30}H_{48}O_3$	$[M-H]^-$	455.3526	0.003	Terpene
85	Bovinic acid[#]	21.385	+	+	+	+	+	+	$C_{18}H_{32}O_2$	$[M-H]^-$	279.2329	0	Fatty acid
86	Unknown31[#]	22.283	+	+	+	+	+	+	$C_{20}H_{29}NO_2$	$[M-H]^-$	653.4266	-	Unknown

Table S1. Cont.

No.	Compound	RT (min)	Tot al	H	MC	EA	Bu	DW	Formula	Adduct	*m/z* (Da)	Error (mDa)	Class
87	Palmitic acid[#]	22.494	+	+	+	+	+	+	$C_{16}H_{32}O_2$	$[M-H]^-$	255.233	87	Palmitic acid[#]
88	Elaidic acid[#]	22.870	+	+	+	+	+	+	$C_{18}H_{34}O_2$	$[M-H]^-$	281.2486	0	Fatty acid
89	Canrenone[#]	27.463	+	+	+	+	+	+	$C_{22}H_{28}O_3$	$[M-H]^-$	339.1998	0.280	Terpene

[#]In-house MS/MS library and online data base such as GNPS, MASS bank.
[§]J.Ethnophamacol. 2022, 298, 115419: 10.1016/j.jep.2022.115419
[$]J. Chem. 2021, doi.org/10.1155/2021/9570776
[†]Reference standard.
"+" and "-": detected and not detected from chromatograms, respectively.

Table S2. Identification of components from total, H, MC, E, B, and W extracts of twigs of *Ilex rotunda*.

No.	Compound	RT (min)	Total	H	MC	EA	Bu	DW	Formula	Adduct	*m/z* (Da)	Error (mDa)	Class
1	Arabitol#	0.808	+	+	+	+	+	+	$C_5H_{12}O_5$	[M-H]-	151.06122	0.214	Glycerol
2	Glucose§,$	0.809	+	+	+	+	+	+	$C_6H_{12}O_6$	[M-H]-	179.05618	0.092	Glycoside
3	Sucrose$	0.811	+	+	+	+	+	+	$C_{12}H_{22}O_{11}$	[M-H]-	341.1088	0.153	Glycoside
4	2-Methylene-4-oxopentanedioic acid#	1.406	+	+	+	+	+	+	$C_6H_6O_5$	[M-H]-	157.01422	2.3297	Organic acid
5	Hypoxanthine#	1.481	+	+	+	+	+	+	$C_5H_4N_4O$	[M-H]-	135.02992	1.083	Alkaloid
6	Threonic acid#	1.481	+	+	+	+	+	+	$C_4H_8O_5$	[M-H]-	135.02992	1.129	Organic acid
7	D-glyceric acid#	1.486	+	+	+	+	+	+	$C_3H_6O_4$	[M-H]-	105.0193	0.359	Organic acid
8	Inosine#	1.500	+	-	+	+	+	+	$C_{10}H_{12}N_4O_5$	[M-H]-	267.0723	1.19	Alkaloid
9	Acetosyringone#	1.554	+	+	+	+	+	+	$C_{10}H_{12}O_4$	[M-H]-	195.06628	0	Phenolic
10	Phthalic acid#	1.560	+	+	+	+	+	+	$C_8H_6O_4$	[M-H]-	165.01939	0.061	Phenolic
11	Succinic acid#	1.600	+	+	+	+	+	+	$C_4H_6O_4$	[M-H]-	117.01934	0.656	Organic acid
12	Adipic acid#	1.653	+	+	+	+	+	+	$C_6H_{10}O_4$	[M-H]-	145.05067	0.076	Organic acid
13	Methylsuccinic acid#	1.865	+	+	+	+	+	+	$C_5H_8O_4$	[M-H]-	131.03499	0	Organic acid
14	(9E)-11a-hydroxy-3,6,10-trimethyl-6,7,8,11-tetrahydro-4H-cyclodeca[b]furan-2,5-dione#	1.915	+	+	+	+	+	+	$C_{15}H_{20}O_4$	[M-H]-	263.11365	4.913	Terpene
15	Unknown3#	2.911	+	+	+	+	+	+	$C_{11}H_{16}O_8$	[M-H]-	275.0774	-	Unknown
16	2,4,5-Trimethoxybenzoic acid#	3.033	+	+	+	+	+	+	$C_{10}H_{12}O_5$	[M-H]-	211.06123	0.229	Phenolic
17	Vanillin#	3.047	+	+	+	+	+	+	$C_8H_8O_3$	[M-H]-	151.0401	0.031	Phenolic
18	Azelaic acid#	3.242	+	-	-	+	+	+	$C_9H_{16}O_4$	[M-H]-	187.09761	0.793	Fatty acid
19	7,8-dimethylalloxazine#	3.575	+	+	+	+	+	+	$C_{12}H_{10}N_4O_2$	[M-H]-	241.07178	1.328	Alkaloid

Table S2. Cont.

No.	Compound	RT (min)	Total	H	MC	EA	Bu	DW	Formula	Adduct	*m/z* (Da)	Error (mDa)	Class
20	2-(Hydroxymethyl)-3-[3,4,5-trihydroxy-6-(hydroxymethyl)oxan-2-yl]oxypyran-4-one#	3.901	+	-	-	+	+	+	$C_{12}H_{16}O_9$	[M-H]-	303.0722	0.0795	Glycoside
21	2-Methoxycinnamic acid#	4.575	+	+	+	+	+	+	$C_{10}H_8O_3$	[M-H]-	177.05573	0	Phenolic
22	Ferulic acid#	4.588	+	+	+	+	+	+	$C_{10}H_{12}O_4$	[M-H]-	195.0663	0.0323	Phenolic
23	(2*R*,3*S*,4*S*,5*R*,6*S*)-2-[(3,4,5-trihydroxyoxan-2-yl)oxymethyl]-6-(3,4,5-trimethoxyphenoxy)oxane-3,4,5-triol#	4.755	+	-	-	+	+	+	$C_{20}H_{30}O_{13}$	[M-H]-	477.16113	0.214	Glycoside
24	3-Methyladipic acid#	4.777	+	+	+	+	+	+	$C_7H_{12}O_4$	[M-H]-	159.06625	0.046	Fatty acid
25	4-Hydroxyphenyllactic acid#	4.779	+	+	+	+	+	+	$C_9H_{10}O_4$	[M-H]-	181.04825	2.35	Phenolic
26	Vanillic acid#	4.910	+	+	+	+	+	+	$C_8H_8O_4$	[M-H]-	167.03497	0.031	Phenolic
27	Syringic acid#	5.280	+	+	+	+	+	+	$C_9H_{10}O_5$	[M-H]-	197.0455	0	Phenolic
28	Genipin#	5.682	+	+	+	+	+	+	$C_{11}H_{14}O_5$	[M-H]-	225.07683	0.031	Glycoside
29	Benzoic acid#	5.700	+	+	+	+	+	+	$C_7H_6O_2$	[M-H]-	121.0295	0	Phenolic
30	Octyl gallate#	5.778	+	+	+	+	+	+	$C_{15}H_{22}O_5$	[M-H]-	281.13934	0	Phenolic
31	Octahydro-6-hydroxy-7-methyl-1-oxocyclopenta[c]pyran-4-carboxylic acid#	6.129	+	+	+	+	+	+	$C_{11}H_{16}O_5$	[M-H]-	227.09253	0.0779	Organic acid
32	Unknown4#	6.369	+	-	+	+	+	+	$C_{20}H_{30}O_{10}$	[M-H]-	429.17633	-	Unknown
33	2-Oxodipic acid#	6.373	+	-	+	+	+	+	$C_6H_8O_5$	[M-H]-	159.02995	0.946	Organic acid
34	3-Carboxy-4-methyl-5-propyl-2-furanpropionic acid#	6.434	+	+	+	+	+	+	$C_{12}H_{16}O_5$	[M-H]-	239.09253	0	Organic acid
35	4,6,8-Trihydroxy-7-methoxy-3-methyl-3,4-dihydroisochromen-1-one#	6.509	+	+	+	+	+	+	$C_{11}H_{12}O_6$	[M-H]-	239.0561	0.015	Phenolic

Table S2. Cont.

No.	Compound	RT (min)	Total	H	MC	EA	Bu	DW	Formula	Adduct	*m/z* (Da)	Error (mDa)	Class
36	4-Vinylphenol[#]	6.669	+	+	+	+	+	+	C_8H_8O	[M-H]-	119.05025	1.152	Phenolic
37	3-Methylbenzoic acid[#]	6.893	+	+	+	+	+	+	$C_8H_8O_2$	[M-H]-	135.0452	0.076	Phenolic
38	*p*-Coumaric acid[$]	6.895	+	+	+	+	+	+	$C_9H_8O_3$	[M-H]-	163.040008	0	Phenolic
39	Cinnamic acid[#]	7.099	+	+	+	+	+	+	$C_9H_8O_2$	[M-H]-	147.04518	0.183	Phenolic
40	Tropic acid[#]	7.116	+	+	+	+	+	+	$C_9H_{10}O_3$	[M-H]-	165.05574	0.015	Phenolic
41	2-Hydroxyoctanoic acid[#]	7.130	+	+	+	+	+	+	$C_8H_{16}O_3$	[M-H]-	159.10271	0.046	Fatty acid
42	Zhebeiresinol[§]	7.401	+	+	+	+	+	+	$C_{14}H_{16}O_6$	[M-H]-	279.0874	0.0821	Phenolic
43	Citrinin[#]	7.401	+	+	+	+	+	+	$C_{13}H_{14}O_5$	[M-H]-	249.07686	0.015	Phenolic
44	Icariside F2[$]	7.835	+	+	+	+	+	+	$C_{18}H_{26}O_{10}$	[M-H]-	425.14514	2.96	Glycoside
45	2-Hydroxy-4-(hydroxymethyl)-6-(1-hydroxy-3-methylbut-2-enyl)-3-[(*E*)-prop-1-enyl]-7-oxabicyclo[4.1.0]hept-3-en-5-one[#]	7.916	+	+	+	+	+	+	$C_{15}H_{20}O_5$	[M-H]-	279.12384	0.061	Terpene
46	9-(2,3-Dihydroxypropoxy)-9-oxononanoic acid[#]	8.010	+	+	+	+	+	+	$C_{12}H_{22}O_6$	[M-H]-	261.1344	0.031	Fatty acid
47	(+)-Syringaresinol *β*-D-glucoside[#]	8.054	+	+	+	+	+	+	$C_{28}H_{36}O_{13}$	[M-H]-	579.20813	1.099	Phenolic
48	(1*S*,4*S*,7*S*,7*S*)-7-hydroxy-7-methyl-1-[(2*S*,3*R*,4*S*,5*S*,6*R*)-3,4,5-trihydroxy-6-[(4-hydroxybenzoyl)oxymethyl]oxan-2-yl]oxy-4,5,6,7-tetrahydro-1H-cyclopenta[c]pyran-4-carboxylic acid[#]	8.106	+	-	+	-	+	+	$C_{23}H_{28}O_{12}$	[M-H]-	495.15067	0.122	Organic acid
49	Melampyroside[#]	8.109							$C_{22}H_{26}O_{10}$	[M+Na]+	473.14190	0	Iridoid
50	6-*O*-Pentopyranosyl-1-*O*-[(2,6,6-trimethyl-1-cyclohexen-1-yl)carbonyl]-*β*-D-glucopyranose[#]	8.257	+	-	+	+	+	+	$C_{21}H_{34}O_{11}$	[M+HCOO]-	507.20816	0.153	Glycoside

Table S2. Cont.

No.	Compound	RT (min)	Total	H	MC	EA	Bu	DW	Formula	Adduct	*m/z* (Da)	Error (mDa)	Class
51	Sinapaldehyde[§,$]	8.483	+	+	+	+	+	+	$C_{11}H_{12}O_4$	[M-H]-	207.06631	0.031	Phenolic
52	Sulfamethoxazole[#]	8.485	+	+	+	+	+	+	$C_{10}H_{11}N_3O_3S$	[M-H]-	252.05145	0	Phenolic
53	3-[(1-Carboxyvinyl)oxy]benzoic acid[#]	8.492	+	+	+	+	+	+	$C_{10}H_8O_5$	[M-H]-	207.03009	0.198	Phenolic
54	Salicylic acid[#]	8.531	+	+	+	+	+	+	$C_7H_6O_3$	[M-H]-	137.02443	0.031	Phenolic
55	(*E*)-9-hydroxy-11-(3-hydroxy-5-(1-hydroxypropyl)tetrahydrofuran-2-yl)undec-10-enoic acid[#]	8.611	+	+	+	+	+	+	$C_{18}H_{32}O_6$	[M-H]-	343.21246	0.275	Phenolic
56	N-Methyltryptamine[#]	8.718	+	+	+	+	+	+	$C_9H_{18}O_3$	[M-H]-	173.11824	0.076	Alkaloid
57	Secoisolariciresinol[#]	8.852	+	+	+	+	+	+	$C_{20}H_{26}O_6$	[M-H]-	407.17096	0.183	Phenolic
58	4-Nitrophenol[#]	8.888	+	+	+	+	+	+	$C_6H_5NO_3$	[M-H]-	138.01968	0.015	Phenolic
59	1-(3,4-Dihydroxy-5-methoxyphenyl)-7-(3,4-dihydroxyphenyl)heptan-3-one[#]	9.137	+	+	+	+	+	+	$C_{20}H_{24}O_6$	[M-H]-	359.14987	0.122	Phenolic
60	4,7,8-Trimethoxy-3,5-dimethylchromen-2-one[#]	9.190	+	+	+	+	+	+	$C_{14}H_{16}O_5$	[M-H]-	263.09256	0.061	Phenolic
61	(2*R*,3*R*,4*R*,6*R*,6*S*,8*S*,11*R*,12*R*,12*S*,14*R*)-2,3,12-trihydroxy-4,6a,6b,11,12,14-hexamethyl-8-[(2*S*,3*R*,4*S*,5*S*,6*R*)-3,4,5-trihydroxy-6-(hydroxymethyl)oxan-2-yl]oxycarbonyl-1,2,3,4a,5,6,7,8,9,10,11,12a,14,14a-tetradecahydropicene-4-carboxylic acid[#]	9.197	+	+	-	+	+	+	$C_{36}H_{56}O_{12}$	[M-H]-	679.36987	0	Terpene
62	Abscisic acid[#]	9.750	+	+	+	+	+	+	$C_{15}H_{20}O_4$	[M-H]-	263.12888	0	Terpene
63	[(6*Z*,10*Z*)-6-(acetyloxymethyl)-10-(hydroperoxymethyl)-3-methylidene-2-oxo-3a,4,5,8,9,11a-hexahydrocyclodeca[b]furan-4-yl] 2-methylbutanoate[#]	9.985	+	+	+	+	+	+	$C_{22}H_{30}O_8$	[M-H]-	421.18649	0.275	Fatty acid

64	Syringaresinol[§]	10.070	+	+	+	+	+	+	$C_{22}H_{26}O_8$	[M-H]-	417.15527	0.214	Phenolic

Table S2. Cont.

No.	Compound	RT (min)	Total	H	MC	EA	Bu	DW	Formula	Adduct	*m/z* (Da)	Error (mDa)	Class
65	5,6,7-Trimethoxy-3-(3,4,5-trimethoxyphenyl)chromen-4-one[#]	10.073	+	+	+	+	+	+	$C_{21}H_{22}O_8$	[M-H]-	401.12402	0.153	Phenolic
66	(1*S*,4*S*,7*S*,7*S*)-7-hydroxy-7-methyl-1-[(2S,3R,4S,5S,6R)-3,4,5-trihydroxy-6-[(4-hydroxybenzoyl)oxymethyl]oxan-2-yl]oxy-4a,5,6,7a-tetrahydro-1H-cyclopenta[c]pyran-4-carboxylic acid[#]	10.073	+	+	+	+	+	+	$C_{23}H_{28}O_{12}$	[M-H]-	495.1507	0.092	Terpene
67	9-Oxononanoic acid[#]	10.074	+	+	+	+	+	+	$C_9H_{16}O_3$	[M-H]-	171.10268	0.87	Fatty acid
68	(2*R*,3*R*)-3,5-Dihydroxy-2-(4-hydroxyphenyl)-7-(3-methylbut-2-enoxy)-2,3-dihydrochromen-4-one[#]	10.212	+	+	+	-	+	+	$C_{20}H_{20}O_6$	[M-H]-	355.11859	0.397	Phenolic
69	3,4-Dimethoxycinnamic acid[#]	10.342	+	+	+	+	+	+	$C_{11}H_{12}O_4$	[M-H]-	207.06631	0.031	Phenolic
70	(10*E*,15*Z*)-9,12,13-Trihydroxyoctadeca-10,15-dienoic acid[#]	10.826	+	+	+	+	+	+	$C_{18}H_{32}O_5$	[M-H]-	327.21768	0.122	Fatty acid
71	2α,3β,23-Trihydroxyolean-12-en-28-oic acid β-D-glucopyranosyl ester[#]	10.939	+	+	+	+	+	+	$C_{36}H_{58}O_{10}$	[M-H]-	695.40106	0.061	Glycoside
72	(*Z*)-9,12,13-trihydroxyoctadec-15-enoic acid[#]	11.257	+	+	+	+	+	+	$C_{18}H_{34}O_5$	[M-H]-	329.23331	0.305	Fatty acid
73	Corosin[#]	11.444	+	+	+	+	+	+	$C_{30}H_{46}O_7$	[M-H]-	517.31683	0.1828	Terpene
74	(4a*S*,6a*S*,6b*R*,9*R*,10*R*,11*R*,12a*R*)-10,11-dihydroxy-9-(hydroxymethyl)-2,2,6a,6b,9,12a-hexamethyl-1,3,4,5,6,6a,7,8,8a,10,11,12,13,14b-tetradecahydropicene-4a-carboxylic acid[#]	11.461	+	+	+	+	+	+	$C_{36}H_{58}O_{10}$	[M-H]-	487.34274	0.183	Terpene

Table S2. Cont.

No.	Compound	RT (min)	Total	H	MC	EA	Bu	DW	Formula	Adduct	*m/z* (Da)	Error (mDa)	Class
75	(4a*S*,6a*S*,6b*R*,8*R*,9*R*,10*R*,11*R*,12a*R*,14b*S*)-8,10,11-trihydroxy-9-(hydroxymethyl)-2,2,6a,6b,9,12a-hexamethyl-1,3,4,5,6,6a,7,8,8a,10,11,12,13,14b-tetradecahydropicene-4a-carboxylic acid[#]	11.593	+	+	+	+	+	+	$C_{30}H_{48}O_6$	[M-H]-	503.33774	0.061	Terpene
76	1-*O*-[(2α,3β,5xi,9xi,18xi)-2,3,23-Trihydroxy-28-oxoolean-12-en-28-yl]-β-D-glucopyranose[#]	11.608	+	+	+	+	+	+	$C_{36}H_{58}O_{10}$	[M-H]-	695.40106	0.061	Glycoside
77	Chrysanthemic Acid[#]	12.401	+	+	+	+	+	+	$C_{10}H_{16}O_2$	[M-H]-	167.10777	0.031	Organic acid
78	Unknown5[#]	12.401	+	+	+	+	+	+	$C_{11}H_{16}O_4$	[M-H]-	211.09758	-	Unknown
79	3β,4β,23-Trihydroxy-24-nor-olean-12-en-28-oic acid[#]	12.730	+	+	+	+	+	+	$C_{29}H_{46}O_5$	[M-H]-	473.3269	0.2218	Terpene
80	3,12-dihydroxy-4,6a,6b,11,12,14b-hexamethyl-1,2,3,4a,5,6,7,8,9,10,11,12a,14,14a-tetradecahydropicene-4,8a-dicarboxylic acid[#]	12.840	+	+	+	+	+	+	$C_{30}H_{46}O_6$	[M-H]-	501.32196	0.031	Terpene
81	Asiatic Acid[#]	13.095	+	+	+	+	+	+	$C_{30}H_{48}O_5$	[M-H]-	487.342651	0.092	Terpene
82	(1*R*,2*R*,4a*S*,6a*R*,6a*S*,6b*R*,8a*R*,9*S*,10*R*,12a*R*,14b*S*)-1,10-dihydroxy-9-(hydroxymethyl)-1,2,6a,6b,9,12a-hexamethyl-2,3,4,5,6,6a,7,8,8a,10,11,12,13,14b-tetradecahydropicene-4a-carboxylic acid[#]	13.280	+	+	+	+	+	+	$C_{30}H_{48}O_5$	[M-H]-	487.3427	0.0942	Terpene
83	Rotungenic acid[§]	13.330	+	+	+	+	+	+	$C_{30}H_{48}O_5$	[M-H]-	487.3428	0	Terpene
84	Octadecanedioic acid[#]	13.338	+	+	+	+	+	+	$C_{18}H_{34}O_4$	[M-H]-	313.23843	0.153	Fatty acid
85	FA 18:4+2O[#]	13.529	+	+	+	-	+	+	$C_{18}H_{28}O_4$	[M-H]-	307.19159	0.671	Fatty acid
86	Astrantiagenin J[#]	13.900	+	+	+	+	+	+	$C_{30}H_{46}O_5$	[M-H]-	485.3266	0.885	Terpene
87	Quillaic acid[#]	14.340	+	+	+	+	+	+	$C_{30}H_{46}O_5$	[M-H]-	485.3268	0.183	Terpene
88	(2α,3β,19α)-2,3,19-TrihydroxyoleanA-12-en-28-oic acid[#]	14.360	+	+	+	+	+	+	$C_{30}H_{48}O_5$	[M-H]-	487.3427	0.58	Terpene

Table S2. Cont.

No.	Compound	RT (min)	Total	H	MC	EA	Bu	DW	Formula	Adduct	*m/z* (Da)	Error (mDa)	Class
89	Euscaphic acid#	14.467	+	+	+	+	+	+	$C_{30}H_{48}O_5$	[M-H]-	487.3423	0	Terpene
90	(*E*,6*S*)-7-hydroxy-2-methyl-6-[(10*S*,13*S*,14*S*,17*S*)-4,4,10,13,14-pentamethyl-3-oxo-1,2,5,6,7,11,12,15,16,17-decahydrocyclopenta[a]phenanthren-17-yl]hept-2-enoic acid#	15.149	+	+	+	+	+	+	$C_{30}H_{46}O_4$	[M-H]-	469.33209	0.214	Terpene
91	9-HOTrE#	15.159	+	+	+	+	+	+	$C_{18}H_{30}O_3$	[M-H]-	293.21222	0.031	Fatty acid
92	13-HpOTrE(r)#	15.304	+	+	+	+	+	+	$C_{18}H_{30}O_4$	[M-H]-	309.20718	0.061	Fatty acid
93	13-HOTrE#	15.347	+	+	+	+	+	+	$C_{18}H_{30}O_3$	[M-H]-	293.21222	0.031	Fatty acid
94	Unknown6#	15.722	+	+	+	+	+	+	$C_{35}H_{54}O_8$	[M-H]-	601.3746	-	Unknown
95	(2*R*,6*R*)-6-[(3*R*,10*S*,12*S*,13*R*,17*R*)-3-(2-carboxyacetyl)oxy-12-hydroxy-4,4,10,13,14-pentamethyl-2,3,5,6,7,11,12,15,16,17-decahydro-1H-cyclopenta[a]phenanthren-17-yl]-2-methyl-3-methylideneheptanoic acid#	15.766	+	-	+	+	+	+	$C_{34}H_{52}O_7$	[M-H]-	571.36383	0.183	Fatty acid
96	Pygenic acid#	15.843	+	+	+	+	+	+	$C_{30}H_{46}O_4$	[M-H]-	471.34765	0.061	Terpene
97	Unknown7#	16.000	+	+	+	+	+	+	$C_{39}H_{52}O_6$	[M-H]-	615.3689	-	Unknown
98	Corosolic acid#	16.032	+	+	+	+	+	+	$C_30H_{48}O_4$	[M-H]-	471.3477	0.366	Terpene
99	Unknown8#	16.117	+	+	+	+	+	+	$C_{38}H_{46}O_6$	[M-H]-	597.322	-	Unknown
100	9-Hode#	16.262	+	+	+	+	+	+	$C_{18}H_{32}O_3$	[M-H]-	295.2278	0.061	Fatty acid
101	Unknown9#	16.320	+	+	+	+	+	+	$C_{47}H_{69}N_3O_9$	[M-H]-	818.49548	-	Unknown
102	Glycyrrhetinic acid#	16.386	+	+	+	+	+	+	$C_{30}H_{46}O_4$	[M-H]-	469.33215	0.183	Terpene

Table S2. Cont.

No.	Compound	RT (min)	Total	H	MC	EA	Bu	DW	Formula	Adduct	*m/z* (Da)	Error (mDa)	Class
103	Unknown10[#]	16.450	+	+	+	+	+	+	$C_{38}H_{46}O_6$	[M-H]-	597.3219	-	Unknown
104	*Trans*-coumaroylcorosolic acid[#]	16.460	+	+	+	+	+	+	$C_{39}H_{54}O_6$	[M-H]-	617.3845	0.1805	Terpene
105	Maslinic acid[#]	16.499	+	+	+	+	+	+	$C_{30}H_{48}O_4$	[M-H]-	471.34769	0.305	Terpene
106	28-*O*-Feruloylbetulin[#]	16.520	+	+	+	+	+	+	$C_{40}H_{58}O_5$	[M-H]-	617.3847	0.068	Terpene
107	(1*S*,2*R*,4a*S*,6a*S*,6b*R*,9*R*,10*R*,11*R*,12a*R*)-10,11-dihydroxy-9-(hydroxymethyl)-1,2,6a,6b,9,12a-hexamethyl-2,3,4,5,6,6a,7,8,8a,10,11,12,13,14b-tetradecahydro-1H-picene-4a-carboxylic acid[#]	16.580	+	+	+	+	+	+	$C_{30}H_{48}O_5$	[M-H]-	487.34268	0.183	Terpene
108	*Trans*-3-feruloylcorosolic acid[#]	16.809	+	+	+	+	+	+	$C_{40}H_{56}O_7$	[M-H]-	647.3951	0.1861	Terpene
109	12,23-trihydroxy-1,6,12,17,23,28-hexazacyclotritriacontane-2,5,13,16,24,27-hexone[#]	17.070	+	+	+	+	+	+	$C_{27}H_{48}N_6O_9$	[M-H]-	599.3875	3.296	Terpene
110	(9*E*)-12-hydroxyoctadec-9-enoic acid[#]	17.072	+	+	+	+	+	+	$C_{18}H_{34}O_3$	[M-H]-	297.2435	0.427	Fatty acid
111	(2*R*,3*R*,4*S*,5*S*,6*R*)-2-[(*E*)-5-[(1*R*,4a*S*,5*S*,6*R*,8a*S*)-6-hydroxy-5,8a-dimethyl-2-methylidene-5-[[(2R,3R,4S,5S,6R)-3,4,5-trihydroxy-6-(hydroxymethyl)oxan-2-yl]oxymethyl]-3,4,4a,6,7,8-hexahydro-1H-naphthalen-1-yl]-3-methylpent-2-enoxy]-6-(hydroxymethyl)oxane-3,4,5-triol[#]	17.094	+	+	+	+	+	+	$C_{32}H_{54}O_{13}$	[M-H]-	645.3432	5.92	Terpene
112	Gypsogenic acid[#]	17.312	+	+	+	+	+	+	$C_{30}H_{46}O_5$	[M-H]-	485.327	0.2523	Terpene
113	Lichesterylic acid[#]	17.334	+	+	+	+	+	+	$C_{18}H_{34}O_3$	[M-H]-	297.2435	2.502	Fatty acid
114	13-Hydroxy-9,11-octadecadienonic acid[#]	17.489	+	+	+	+	+	+	$C_{18}H_{32}O_3$	[M-H]-	295.2278	0	Fatty acid
115	13*S*-Hydroxyoctadecadienoic acid[#]	17.630	+	+	+	+	+	+	$C_{18}H_{32}O_3$	[M-H]-	295.2279	0.0063	Fatty acid

Table S2. Cont.

No.	Compound	RT (min)	Total	H	MC	EA	Bu	DW	Formula	Adduct	*m/z* (Da)	Error (mDa)	Class
116	Avenoleic acid[#]	17.821	+	+	+	+	+	+	$C_{18}H_{32}O_3$	[M-H]-	295.2278	0.0063	Fatty acid
117	Hederagenin[#]	18.202	+	+	+	+	+	+	$C_{30}H_{46}O_4$	[M-H]-	471.34769	0.2968	Terpene
118	Linolenic acid[#]	20.038	+	+	+	+	+	+	$C_{18}H_{30}O_2$	[M-H]-	277.21729	0.916	Fatty acid
119	Unknown11[#]	20.048	+	+	+	+	+	+	$C_{39}H_{54}O_6$	[M-H]-	617.38458	-	Unknown
120	(4a*S*,6a*S*,6b*R*,10*S*,12a*R*)-10-hydroxy-2,2,6a,6b,9,9,12a-heptamethyl-1,3,4,5,6,6a,7,8,8a,10,11,12,13,14b-tetradecahydropicene-4a-carboxylic acid[#]	20.330	+	+	+	+	+	+	$C_{30}H_{48}O_3$	[M-H]-	455.35263	0.305	Terpene
121	Ursolic acid[§,$]	20.707	+	+	+	+	+	+	$C_{30}H_{48}O_3$	[M-H]-	455.352	1.526	Terpene
122	Unknown12[#]	21.293	+	+	+	+	+	+	$C_{39}H_{48}O_6$	[M-H]-	611.33765	-	Unknown
123	Linoelaidic acid[#]	21.384	+	+	+	+	+	+	$C_{18}H_{32}O_2$	[M-H]-	279.23294	0.305	Fatty acid
124	Unknown13[#]	21.812	+	+	+	+	+	+	$C_{36}H_{66}O_7$	[M-H]-	609.47345	-	Unknown
125	Unknown14[#]	22.284	+	+	+	+	+	+	$C_{36}H_{62}O_{10}$	[M-H]-	653.42688	-	Unknown
126	Palmitic Acid[#]	22.464	+	+	+	+	+	+	$C_{16}H_{32}O_2$	[M-H]-	255.2328	0.183	Fatty acid
127	Oleic Acid[#]	22.863	+	+	+	+	+	+	$C_{18}H_{34}O_2$	[M-H]-	281.2486	0.061	Fatty acid
128	Stearic Acid[#]	24.427	+	+	+	+	+	+	$C_{18}H_{36}O_2$	[M-H]-	283.2642	0.366	Fatty acid

[#]In-house MS/MS library and online data base such as GNPS, MASS bank.
[§]J.Ethnophamacol. 2022, 298, 115419: 10.1016/j.jep.2022.115419
[$]J. Chem. 2021, doi.org/10.1155/2021/9570776
[†]Reference standard.
"+" and "-": detected and not detected from chromatograms, respectively.

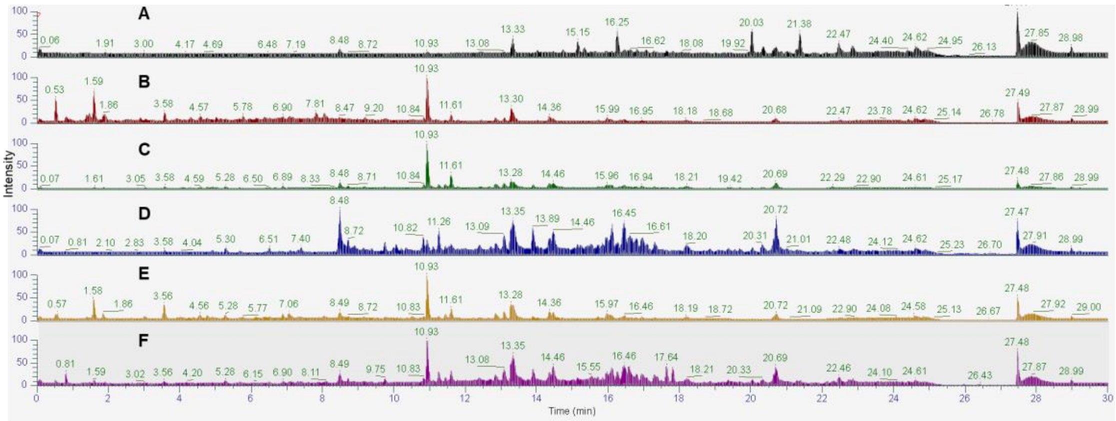

Figure S1. Chromatograms of H (A, black), E (B, red), B (C, green), MC (D, blue), W (E, yellow) and extract (F, magenta) of twigs of *I. rotunda* detected in TIC of negative ion mode.

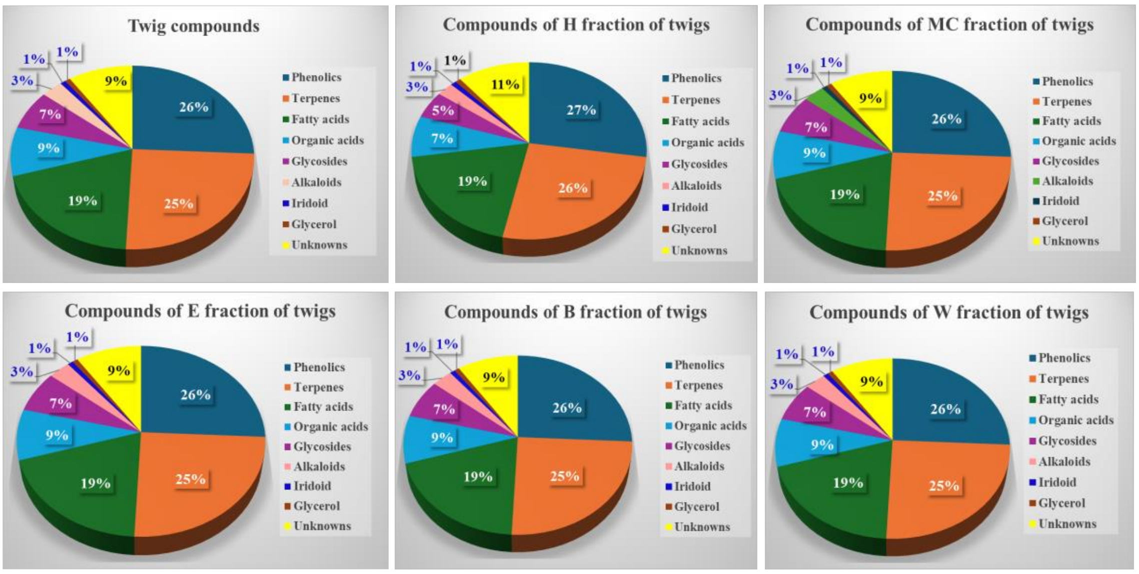

Figure S2. Composition of compounds from the extract and fractions of twigs of *I. rotunda*.

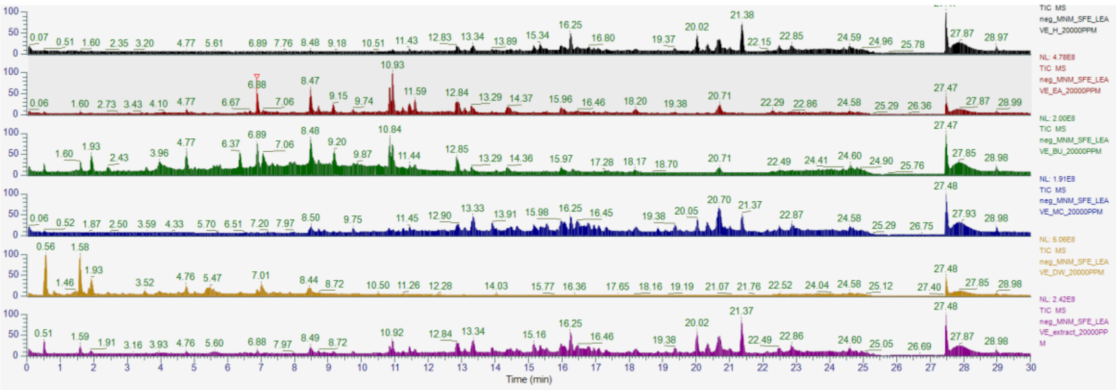

Figure S3. Chromatograms of H (A, black), E (B, red), B (C, green), MC (D, blue), W (E, yellow) and extract (F, magenta) of leaves of *I. rotunda* detected in TIC of negative ion mode.

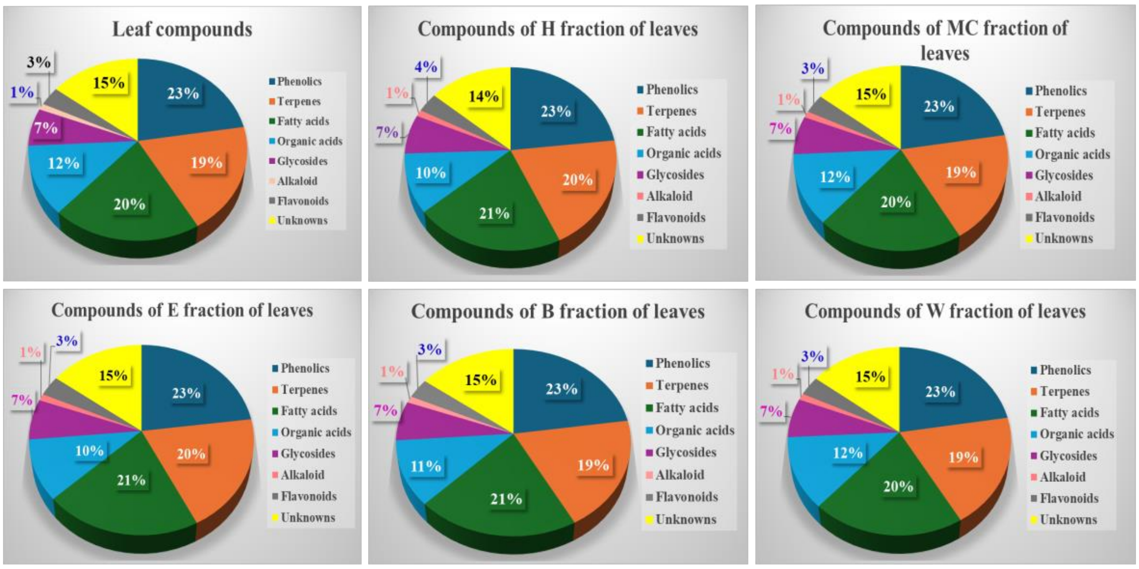

Figure S4. Composition of compounds from the extract and fractions of leaves of *I. rotunda*.
